# Unfolded Protein Response and Macroautophagy in Alzheimer’s, Parkinson’s and Prion Diseases

**DOI:** 10.3390/molecules201219865

**Published:** 2015-12-18

**Authors:** Irina Milisav, Dušan Šuput, Samo Ribarič

**Affiliations:** 1 Institute of Pathophysiology, Faculty of Medicine, Zaloška 4, Ljubljana SI-1000, Slovenia; irina.milisav@mf.uni-lj.si (I.M.); dusan.suput@mf.uni-lj.si (D.S.); 2 Faculty of Health Sciences, Zdravstvena pot 5, SI-1000 Ljubljana, Slovenija

**Keywords:** Alzheimer’s disease, autophagy, neurodegeneration, Parkinson’s disease, prion diseases, proteostasis, unfolded protein response

## Abstract

Proteostasis are integrated biological pathways within cells that control synthesis, folding, trafficking and degradation of proteins. The absence of cell division makes brain proteostasis susceptible to age-related changes and neurodegeneration. Two key processes involved in sustaining normal brain proteostasis are the unfolded protein response and autophagy. Alzheimer’s disease (AD), Parkinson’s disease (PD) and prion diseases (PrDs) have different clinical manifestations of neurodegeneration, however, all share an accumulation of misfolded pathological proteins associated with perturbations in unfolded protein response and macroautophagy. While both the unfolded protein response and macroautophagy play an important role in the prevention and attenuation of AD and PD progression, only macroautophagy seems to play an important role in the development of PrDs. Macroautophagy and unfolded protein response can be modulated by pharmacological interventions. However, further research is necessary to better understand the regulatory pathways of both processes in health and neurodegeneration to be able to develop new therapeutic interventions.

## 1. Proteostasis in Neurodegeneration

**Figure 1:**
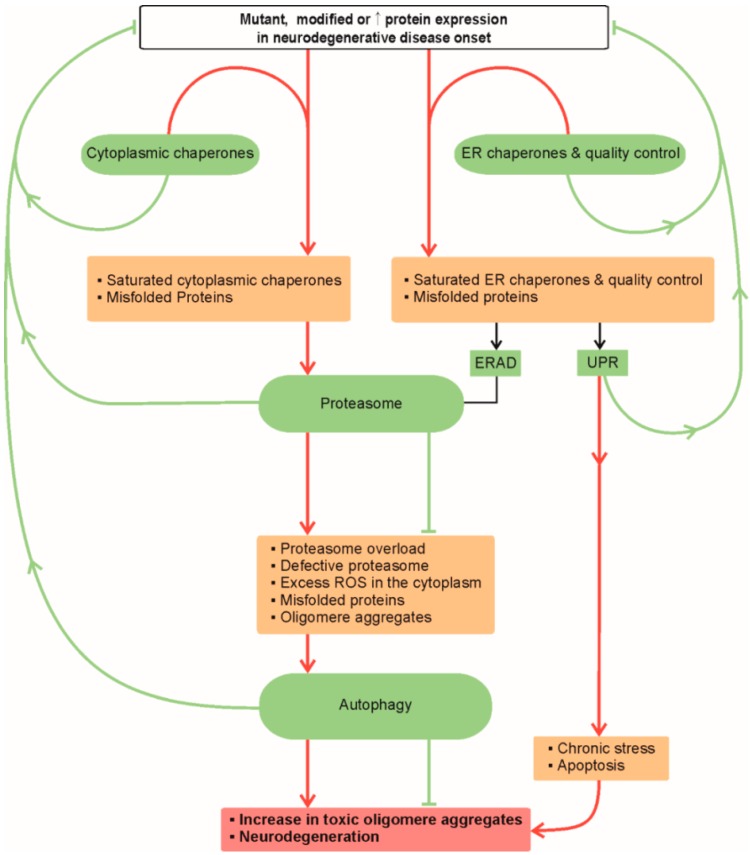
Proteostasis in neurodegeneration. Red lines and arrows indicate progressive failure of proteostasis ultimately leading to neurodegeneration. Green arrows and lines indicate appropriate response of proteostasis to altered proteins that prevents or slow-downs the progress of neurodegeneration. Abbreviations: ER (endoplasmic reticulum); ERAD (endoplasmic reticulum associated protein degradation); ROS (reactive oxygen species); UPR (unfolded protein response).

There are integrated biological pathways within cells that control the protein synthesis, folding, trafficking and degradation of proteins referred to as protein homeostasis or proteostasis. There are dynamic interconnections between the cytosolic and endoplasmic reticulum protein syntheses, quality control, folding and degradation mechanisms in neurons. Protein folding capacity may become saturated in the stages prior to neurodegeneration; e.g., due to large amounts of misfolded proteins, protein aggregation and mutant proteins (Figure 1). Oversaturation of both, cytoplasmic and endoplasmic reticulum (ER) protein synthesis and folding pathways, is rescued by proteasomal degradation and autophagy of unfolded, misfolded, mutant or damaged (e.g., oxidised) proteins. In addition to the ER associated protein degradation pathway (ERAD), the oversaturation of ER triggers also an unfolded protein response (UPR) to reduce the load of newly synthesized proteins temporarily. If these mechanisms do not manage to clear the aberrant proteins, if there is a proteasome overload, proteasome is defective or there are excess ROS in the cytoplasm, misfolded proteins may form aggregates and the UPR mechanism is extended. These aggregates are attempted to be cleared by macroautophagy. The further inefficient clearance of aggregates, formation of toxic aggregates and prolonged oxidative stress further inhibit the proteasome and ERAD, resulting in chronic stress and apoptosis that may be triggered by the UPR [1].

Proteostasis in the brain is more susceptible to perturbations than in other organs for the following reasons: (a) an increased susceptibility of neurons even to a brief episode of hypoxia/ischemia due to the dependence on a continuous supply of oxygen and glucose; (b) the absence of cell division that prevents replacement of damaged neurons after apoptosis or necrosis and leads to an increased exposure of nerve cells to age-related changes of proteostasis [2,3]; (c) effects of chronic disorders (e.g., protein glycation in diabetes); and (d) the high turnover of cell components involved in synaptic transmission coupled with the high metabolic rate at the synapses.

Alzheimer’s disease (AD), Parkinson’s disease (PD) and prion diseases (PrDs) have different clinical manifestations of neurodegeneration, however, all share an accumulation of misfolded pathological proteins associated with perturbations in unfolded protein response [1,4], and macroautophagy [5,6]. This paper will focus on the role of unfolded protein response and macroautophagy in AD, PD and PrDs as examples of perturbed proteostasis in neurodegenerative disorders.

### 1.1. Endoplasmic Reticulum

One of the key functions of the ER, composed of double membrane sheets adjacent and continuous with the outer layer of the nuclear envelope, is the synthesis, folding and quality control of cell membrane proteins. ER and Golgi (G) work in concert to target newly synthetized proteins to their final destination [1]. In contrast to the continuous connection between the nucleus and ER, the bi-directional transport between ER and G is enabled by membrane bound vesicles. The coating proteins for these vesicles are COPI and COPII. COPI targets vesicles to the G, and COPII enables retrograde transport [7]. The shuttle of these transport vesicles is enabled by molecular motors and the cytoskeleton, a flexible cytosolic network that rapidly responds to regional requirements by adjusting its assembly/disassembly ratio. A key component of human and animal cells’ cytoskeleton are microtubules, made of polymerised tubulin. Some neurodegenerative disorders, e.g., PD and AD, are associated with changes in tubulin polymerisation [8,9].

**Figure 2:**
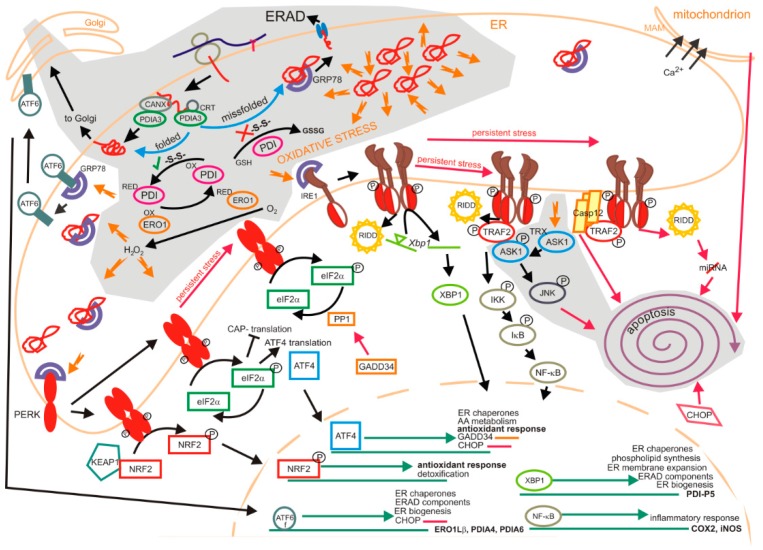
Basic mechanisms of unfolded protein response. There are three phases of UPR. The adaptive, transition (black arrows) and the late phase (red arrows); the last two phases occur if the stress is not resolved and there is prolonged stress. Protein load in the ER is decreased during the early adaptive phase. ATF6, PERK and IRE1 are the three classes of main ER stress sensors that respond to the levels of unfolded/misfolded proteins in the ER. They are activated by the dissociation of GRP78/BiP upon increased amounts of misfolded proteins or direct binding of unfolded proteins in the case of yeast Ire1p and possibly IRE1β. Then the cell survival or cell death pathways are conveyed through complex parallel and convergent signal transduction pathways. Impact of oxidative stress on protein folding and unfolded protein response is depicted on the grey background. Protein disulphate isomerases (PDIs) assist in folding of nascent chains by catalization of disulphide bond formation (top left). For example, PDIA3 or Erp57 is expressed in many tissues, including liver, pancreas, kidney, placenta and lungs and to lower extent in heart, skeletal muscle and brain.. Its activity is enhanced when it is in complex with calnexin (CANX) and calreticulin (CRT). The proper formation of disulphide bonds between two cysteine residues in nascent proteins is necessary for correct protein folding. Two cysteines in the active site of PDI accept two electrons from the cysteines of the folding polypeptide. PDI is then oxidized by oxidoreduction 1 (ERO1) proteins that subsequently transfer the electrons to oxygen to produce H_2_O_2_. Improperly paired disulphide bonds are reduced by PDI, while producing the oxidized glutathione. Misfolded proteins are exported to the cytosol to be degraded by the proteasome in the process ER-associated degradation (ERAD). ROS produced during protein folding and the decrease of GSH upon the reduction of improperly paired disulphide bonds of misfolded proteins may shift the redox balance towards the oxidative stress. Likewise, oxidative stress is the consequence of excess of misfolded proteins. Upon oxidative stress, all three ER stress sensors, ATF6, PERK and IRE1α are activated. Apoptosis signal-regulating kinase (ASK1) is activated by oxidative stress, ER stress and inflammation (e.g., TNFα). It dissociates from (oxidized) thioredoxin (Trx) and binds TRAF2 to convey the apoptosis signal through JNK activation.

Discrete sites of ER, the mitochondria-associated ER membranes or MAMS (Figure 2), are intimately associated with mitochondria, enabling for example, membrane lipids, lipid, Ca^2+^ and ROS transfer to the mitochondria, mitochondrial fission, inflammasome formation and preparation of mitochondrial membranes for autophagy; for a review see [10]. The PKR-like ER kinase (PERK) is important for the integrity of the MAMs contact points [11]. An increased presence of proteins, e.g., Aβ and presenilin 2, increase the number of MAMs [12,13]. ER is the major Ca^2+^ storage site in the cell. The net flux of Ca^2+^, from ER to mitochondria, increased by ER stress, is due to the interaction between inositol 1,4,5-trisphosphate) receptor (IP3R) and cytochrome c. Cytochome c blocks the Ca^2+^ dependent inhibition of IP3R, leading to an Ca^2+^ efflux from the ER and cell wide cytochrome c release result in apoptosis [12,13,14,15,16].

Protein folding is enabled by several ER chaperone proteins (e.g., 78 kDa glucose-regulated protein, GRP78/BiP; protein disulphate isomerase, PDI) and is essential for the transport of newly synthetized proteins from ER to G. Misfolded proteins or proteins that can’t be folded are targeted for degradation by the ER-associated protein degradation (ERAD) pathway and returned to the cytosol; they are ubiquitinated by the ER-associated ubiquitin ligases for degradation by the proteasome. The ER responds to different stressors, for example to oxidative stress, overexpression of proteins (e.g., α- synuclein, αSYN; amyloid beta, Aβ), hypoxia/ischemia and insulin resistance, by (a) slowing the rate of protein folding and transiently increasing the content of unfolded proteins; and (b) by an overall translational block and activation of ER stress responsive genes that will increase the protein folding capacity and decrease the protein folding load in the ER [1,4,17].

### 1.2. Unfolded Protein Response

Oxidative stress, increased protein synthesis, decreased ER-associated protein degradation, disturbed calcium signalling and disturbed lipid homeostasis can result in ER stress and activate the unfolded protein response [18]. In mammals there are three classes of ER stress sensors that respond to the levels of unfolded/misfolded proteins in the ER. All of them are ER membrane-associated proteins: (a) activating transcription factor 6, (ATF6); (b) pancreatic ER eIF2α kinase (PERK, also, PKR-like ER kinase, double-stranded RNA-activated protein kinase-like ER kinase); and (c) inositol-requiring kinase 1 (IRE1) (Figure 2) [19]. The luminal domain of each stress sensor is bound to the chaperone GRP78/BiP in the resting state. GRP78/BiP dissociate upon the ER stress to bind unfolded proteins in mammals [19,20]. There are two mammalian IRE1 homologues IRE1α and IRE1β. IRE1α is expressed ubiquitously, while IRE1β is mostly in intestinal epithelium [19,21]. Like Ire1p from yeast, the mammalian IRE1β may bind unfolded proteins directly [22]. Similarly, there are two ATF6 isoforms in mammals; their *N*-terminal cytosolic fragments (ATF6f) are transcription factors. The relative levels of the two ATF6 isoforms may contribute to the regulation of strength and duration of ATF6f dependent ER stress response gene induction [23]. The induced genes have ER stress response elements (ERSE) within their promoter encoding proteins that collectively may reduce ER stress, produce a transcript for pro-survival transcription factor and a target of IRE1, X-box binding protein-1 (XBP1, see below) and of the pro-apoptotic transcription factor C/EBP homologous protein (CHOP) during the sustained ER stress [20,24].

PERK and IRE1 are protein kinases that sense ER stress by its luminal domains, detect alterations in the lipid composition of the ER membrane through their transmembrane regions and propagate downstream events through cytosolic regions [25,26]. Most of the PERK signalling is mediated through phosphorylation of the alpha subunit of eukaryotic initiation translation factor 2 (eIF2α which result in inhibition of delivery of the initiator methionyl-tRNA to the ribosome, resulting in general inhibition of protein translation (cap-dependent translation) to decrease protein influx into ER [27,28]. eIF2α phosphorylation also promotes the translation of selective mRNAs with internal entry ribosomal site (IRES), leading to the translation of genes associated with UPR, namely the transcription factor gene 4 (ATF4) [28,29]. This results in an up-regulation of genes associated with redox homeostasis, energy metabolism and protein folding [30,31]. PERK also phosphorylates other proteins, like NRF2 and NF-κB; this may result in adjustments of redox metabolism and inflammatory processes [25]. Apart from pro-survival signalling pathways, there are also pro-apoptotic ones initiated downstream of PERK and ATF4 that are important during the persistant ER stress and may contribute to neurodegeneration. Growth arrest and DNA damage - inducible 34 (GADD34) regulates the activity of protein phosphatase 1 (PP1) that inactivates eIF2 thus resumes the cap-dependent translation [32] and increases protein load in the ER, while CHOP is the transcription factor, for example needed for transcription of pro-apoptotic BCL2 genes. CHOP expression is induced by ATF4 in combination with other transcription factors [33].

Activated IRE1 is a kinase participating in cell fate determination through mitogen protein kinase (JNK, also c-Jun *N*-terminal kinase) and an endonuclease splicing mRNA for the transcription factor XBP-1 to trigger its translation [21]. XBP-1 modulates expression of UPR target genes, including ER chaperons, glycosylation enzymes, ERAD components and those involved in the synthesis of phospholipids [25]. Other RNAs are targeted through a process called regulated IRE1-dependent decay (RIDD) that reduces the amount of proteins targeted to the ER. In the case of persistent ER stress, IRE1 ceases to splice *Xbp*-*1* mRNA to repress adaptive responses and activate apoptosis through RIDD [21]. RIDD can increase ER stress intensity through degradation of selective UPR target genes like GRP78 [21]. As the ER stress intensity reaches its threshold, IRE1 degrades some microRNAs linked to regulation of apoptosis, among them pre-miRNA for Caspase-2 [21]. Modulation of IRE1 activity depends on its interacting proteins. IRE1 also controls cell fate determination through its kinase activity. The cytosolic domain of IRE1 binds the adaptor protein TNF-receptor-associated factor 2 (TRAF2) that promotes the activation of apoptosis-signal regulating kinase 1 (ASK1) and JNK (c-Jun *N*-terminal kinase), [25]. IRE1 can also modulate other MAP kinases (ERK, p38) [34,35] and NF-κB pathway through interacting with inhibitor κB kinase (IKK) through TRAF2 [36]. CHOP upregulation may be a point of convergence for all 3 arms of UPR, as there are binding sites for ATF6, ATF4 and XBP1 in its promoter [24]. PERK and IRE1 are active throughout the duration of ER stress [21]; there is also a view that ATF6 and PERK may be activated before IRE1α, as they promote adaptational responses. IRE1 has a dual role transmitting both survival and apoptotic signals [24]. Two novel mechanisms that can reduce the protein burden within minutes of ER stress were published in 2014. “Rapid ER stress-induced export” (RESET) removes misfolded GPI-anchored proteins through the secretory pathway for lysosomal degradation [37,38]. The second mechanism is selective release of mRNAs for ER-targeted proteins from the ER membrane upon the ER stress, [39]. The molecular mechanisms of UPR triggering and propagation are depicted in Figure 2.

ER and mitochondria are interconnected physically and functionally by mitochondria-associated ER membranes (MAMs, top right). A sustained ER stress of more than 24 h or the induction of apoptosis results in the release of ER calcium at the MAM that triggers loss of mitochondrial membrane potential to promote apoptosis [40].

In conclusion, UPR signalling is at first aimed at cell survival and it is an adaptive response during the early phases of ER stress [21]. The goals are reduction in the ER protein folding load and increased ER folding capacity [35]. Protein folding capacity is enhanced by expansion in ER volume and by increased synthesis of ER chaperones. Depending on the duration of stress, the UPR signalling changes to repress the adaptive response and trigger apoptosis [21]. Pathophysiological conditions, like elevated levels of fatty acids and cholesterol, high or low glucose levels, inflammatory cytokines and hypoxia can chronically activate the UPR [18]. As UPR signalling is interconnected with oxidative stress and inflammatory response pathways, its persistent signalling in prolonged stress has a role in many diseases, including metabolic and inflammatory diseases, cancer and neurodegeneration [18].

#### 1.2.1. Impact of Oxidation on the Unfolded Protein Response

Oxidative environment in the ER favours formation of disulfide bonds. The proper formation of disulfide bonds between two cysteine residues is necessary for correct protein folding. These reactions are catalized by protein disulfide isomerases (PDIs) that are ubiquitously expressed in the ER [41,42]. The members of PDI family also cleave disulphide bonds of misfolded proteins and catalyze isomerization of incorrect disulfides [41], while abberant proteins are cleared through ER-associated degradation (ERAD) [43]. Most of about 20 members of the PDI family [44,45,46] contain ER retention signal (KDEL) and at least one thioredoxin (Trx)—like catalytic domain. PDI family include PDIA3/ERp57, see Figure 2), ERp44 [47], ERdj5, *etc.* ERdj5 is required for ER export of terminally misfolded proteins in the ERAD; it reduces the disulphide bonds in ERAD substrates [48,49].

Oxidoreductin proteins 1 oxidize PDIs and regenerate themselves by transfering electrons to oxygen to produce H_2_O_2_ [41,42]. Peroxiredoxin IV was reported to metabolize H_2_O_2_ into H_2_O [50]. There are two members of oxidoreductin 1 in mammals, Ero1α and Ero1β, which differ in transcriptional and post-translational regulation, tissue distribution and catalytic turnover [51]. Besides from assisting in disulfide bond generation, Ero1α also regulates calcium release from the ER and secretion of disulfide-linked oligomers through its reversible association with the chaperone ERp44 [51].

Redox status in ER is maintained also by glutathione concentrations. Like elsewhere in the cell, the balance between the reduced and oxidized glutathiones (GSH and GSSG, respectively) is important. The ratio of GSH to GSSG in the ER is 1:1 to 1:3, compared to the 30:1 to 100:1 in the cytosol [52]. The reduction of improperly paired disulfide bonds by PDIs is accompanied by glutathione oxidation (Figure 2). Disruption of redox balance in the ER due to environmental or intrinsic factors can result in loss of ER homeostasis and triggers the UPR due to accumulation of misfolded proteins. Increased incorrect disulfide bond formation during protein folding and their reduction in misfolded proteins result in redox imbalance and oxidative stress. Therefore, there is a close relationship between the oxidative and and ER stresses and control of UPR is also through the regulation of ER redox capacity [53].

The role of transitional metal ions in the development of human disease has been recently reviewed by Valko *et al.* [54]. Copper, iron, and zinc ions, have an important role in various biological processes. Dysregulation of their homeostasis, *i.e.*, oxidative stress, can lead to an increased, uncontrolled formation of reactive oxygen species (ROS) and reactive nitrogen species (RNS) that overwhelms their elimination by antioxidant defence systems and leads to an increased frequency of damage to DNA, protein and lipid molecules. An increase, or a decrease, in the concentration of metal ions can lead to dysregulation of their homeostasis. For example, increased concentrations of redox-active iron or copper ions promote the formation of ROS, while increased ROS formation is associated with depletion of redox-inert zinc ions. Zinc is not only an essential component of proteins involved in defence mechanisms against oxidative stress but is also the most abundant metal in the brain.

#### 1.2.2. Degradation of Oxyproteins by the Unfolded Protein Response

An important mechanism to restore ER proteostasis is the removal of misfolded proteins. Misfolded proteins are exported from the ER before degradation. There are two protein degradation pathways: autophagy and ERAD; the latter delivers the proteins to proteasome. Activation of UPR triggers both pathways [55,56,57]. Autophagy was reported to be the main degradation pathway in neural cells [58]; however, ERAD seems closely linked to the UPR, which has a role in protection and progression of neurodegenerative diseases. Interestingly, impairment of ERAD resulted in decreased UPR activation and protection against the ER stress toxicity in SK-*N*-SH and HeLa cells [59]. The authors reported that there was a modulation of UPR upon the inhibition of ERAD; the response involved also re-localization of lysosomes. Large parts of ER can be sequestered into autophagosomes [56] and particular proteins can be removed by autophagy. The examples of the latter are the removal of ubiquitinated-protein aggregates that develop during diabetes mellitus-induced oxidative stress in pancreatic beta cells [60] and of misfolded GPI-anchored proteins that were recently shown to be removed from the ER through the secretory pathway for lysosomal degradation described in Section 1.2 [32].

ERAD targets the misfolded proteins to the cytosol for the proteasome degradation; such proteins are either in the ER lumen or are ER membrane proteins [43]. Removal of ER proteins involves substrate recognition [43,48,61,62,63,64], dislocation [62,64,65,66,67,68,69], ubiquitination [43,63] and degradation. Although many components that are necessary for ERAD were identified, the knowledge on its core components, the precise molecular mechanism of dislocation as well as the energy source still need some improvement. Even now there are hints of connections between the UPR and ERAD, e.g., the UPR activation increases the levels of ERAD genes, like EDEM1 [70].

#### 1.2.3. Unfolded Protein Response in Neurodegenerative Disorders

UPR is a complex signalling network that may result either in alleviation of ER stress or in cell death. Although many papers have described its key role in normal neuronal function and there are more and more papers implying its role in neurodegeneration, the reports often contradict each other, as the UPR activation seems to have specific and even opposing effects on neurodegeneration. The contributing factors to such dichotomous response seem to depend on the disease stage and include several stress sensors with complex signalling at protein and nucleic acid levels as well as the cross-talk with other cellular stress responses, for review see [1,4]. The role of UPR in Alzheimer’s, Parkinson’s and prion diseases is described in Section 2.2, Section 2.3, Section 3.2, Section 3.3, Section 4.2 and Section 4.3, respectively.

### 1.3. Macroautophagy

Autophagy is essential for maintaining cellular homeostasis and is divided into macroautophagy (MA), microautophagy (MI), and chaperone-mediated autophagy (CMA). Among the three types of autophagy, MA is the best understood process and is often referred to as autophagy. MA is the only known mechanism that eukaryotic cells possess to degrade protein aggregates and superfluous or damaged organelles that cannot be processed by the proteasome [71]. During MA, portions of the cytoplasm are enveloped by a double-membrane sac (*i.e.*, phagophore) that expands into a cytosolic vesicle called an autophagosome. The outer membrane of the completed autophagosome is fused in mammalian cells with lysosomal membrane forming an autolysosome that shares the contents of both and enables lysosomal acid hydrolases access to the inner autophagosome membrane and its cargo, which is digested and recycled by the cell [72]. Autophagosome formation is often monitored by analysing the level of LC3-II which is associated with autophagosome membranes and is degraded after formation of the autolysosome [73].

The uptake of cytoplasmic materials by MI is a two-step process, first by invagination of the lysosome membrane and then by pinch off of the vesicles containing the cytosolic substrates into the lysosomal lumen, where they are rapidly degraded [74]. Proteins targeted for degradation by CMA, for example αSYN or LRRK2, are identified one-by-one by the cytosolic chaperone Hsc70 that delivers them to the surface of the lysosomes [75]. The substrate proteins bind, unfold and enter the lysosome through a receptor-channel on the lysosomal membrane, enabling direct translocation of unfolded proteins across the lysosome membrane [76,77].

In contrast to the ubiquitin-26S proteasome system, MA often mediates non-selective and bulk degradation of cytoplasmic contents, including entire organelles, for example mitochondria (mitophagy) endoplasmic reticulum (reticulophagy) or ribosomes (ribophagy) [78,79,80]. Mitophagy removes dysfunctional mitochondria, thus reducing mitochondria derived ROS that could generate mitochondrial DNA mutations during aging [81]. However, in some cases, MA has substrate specificity, for example in the cytoplasm-to-vacuole-targeting (CVT) pathway where autophagy has a biosynthetic role [82,83,84].

MA is regulated by protein kinases; mTOR inhibits MA, while AMPK and ULK1 promote MA. Downstream regulators of MA are proteins RAB1a, Beclin1 and ATG protein family that promote the initiation of MA [6]. Under normal conditions, the basal MA of brain cells is low compared to other organ systems. However MA, together with ER, significantly contributes to intracellular quality control, especially in post mitotic nerve cells, that are susceptible to accumulation of misfolded or degraded proteins over decades of human life [5,85,86].

Dysregulation of MA contributes to the development and progression of Alzheimer’s, Parkinson’s and prion diseases [5,6,71,87]. In neurodegenerative disorders, there is a significant increase in autophagosomes [88]. When interpreting the presence of an increased number of autophagosomes it is important to distinguish between measurements that evaluate the numbers of autophagosomes and those that measure flux through the MA pathway. Blockage of MA flux, for example due to disturbance in lysosomal function, leads to autophagosome accumulation and has to be distinguished from fully functional MA that includes fusion of autophagosome with lysosomes, autolysosome formation, cargo degradation and recycling [5]. Most studies support the role of MA in preventing or at least attenuating the progression of neurodegenerative disorders. For example, impaired autophagic flux enables accumulation of toxic Aβ [89]. Clearance of aggregate prone forms of αSYN is mediated by MA [90]; pharmacological activation of MA by lithium or trehalose accelerates clearance of aggregate prone A30P and A53T mutants of αSYN [91]. In contrast to PD and AD, where an enhanced MA seems to protect or at least slow down the progression of neurodegenerative disorders, MA seems to have opposing effects in prion diseases. Fibroblasts from Atg5−/− autophagy deficient mice were significantly less infectable with murine prions than the wild-type fibroblasts and re-introduction of *Atg5* via lentiviral transduction improved the infection rate in Atg5−/− mice [5]. In a prion infected cell model, MA reduced the cellular content of the normal form of prion protein (PrP^c^) and the infectious prion protein isoform (PrP^Sc^), MA inhibitors increased cellular PrP^Sc^, and lithium, trehalose and rapamycin reduced the level of cellular PrP^Sc^ presumably by activating MA [5,92,93].

## 2. Alzheimer’s Disease

Alzheimer’s disease (AD) is the most common cause of dementia with significant pathological and clinical diversity among clinically diagnosed AD patients. Most elderly patients have several pathological changes in the brain, as observed in autopsy studies that in addition to the senile plaques also include Lewy bodies, white matter changes, TDP-43 inclusions or angiopathy [94,95,96,97,98,99,100,101]. Biomarker tools for AD are being developed that have enabled detection of AD related pathological brain changes years before emergence of the first clinical symptoms. For example, cerebrospinal levels of tau are positively correlated to neurodegeneration and neurofibrillary tangle pathology, however, cerebrospinal fluid (CSF) levels of the aggregation prone 42 amino acid Aβ (Aβ42) are negatively correlated to plaque pathology. Amyloid plaques and tau can be detected with positron emission tomography [102,103]; the appearance of the Aβ42 marker precedes tau by 5–10 years [104,105]. There is a need for further development of diagnostic markers for AD. A recent Cochrane review concluded that measuring CSF amyloid beta (Aβ40 or Aβ42), as a single test, lacks the accuracy to identify patients with mild cognitive impairment who would develop Alzheimer’s disease dementia or other forms of dementia [106]. AD is associated with systemic manifestations that are present in early as well as in the late stages of this neurodegenerative disorder [107]. Some systemic disorders are associated with an increased risk of developing AD like for example type 2 diabetes [108,109,110] and inflammation [111]. The role of oxidative stress biochemical pathways in the overlapping of some of the AD and type 2 diabetes clinical signs has been recently reviewed by Rosales-Corral *et al.* [112].

### 2.1. Amyloid β (Aβ) and Tau

The key pathological change in Alzheimer’s disease is the modification of soluble amyloid β-peptides (Aβs), for example Aβ42, that accumulate in the intracellular and extracellular space, into toxic, soluble Aβ oligomers leading to cell death [113]. Aβ42 monomer *per se* is not toxic, but has a neuroprotective role mediated by activation of the phosphatidylinositol-3-kinase pathway, and the stimulation of receptors of the insulin superfamily [114]. Aβs are derived by proteolytic cleavage of the type 1 membrane glycoprotein, amyloid precursor protein (APP). The APP is processed by two pathways: (a) the amyloidogenic pathway, where the APP is sequentially cleaved by β- and γ-secretase; and (b) the non-amyloidogenic pathway where the APP is cleaved by α- and then γ-secretase. The non-amyloidogenic pathway produces the soluble sAPPα that have neuroprotective and memory-enhancing effects [115,116]. In physiological conditions, Aβ could present a negative feedback loop in the regulation of synaptic plasticity and neuronal survival since low concentrations of Aβ are present in the central nervous system of non-demented individuals [117].

Amyloid processing of APP is concentrated in lipid rafts, and non-amyloid processing is located mainly in the non-raft regions of the cell membrane [118,119,120,121,122]. In physiological conditions, the majority of APP is processed by the non- amyloidogenic pathway, therefore increased Aβ production correlates with decreased sAPPα levels [123,124,125,126]. Both APP processing pathways are modulated by various factors; some metalloproteases have α secretase activity and could contribute to the production of soluble sAPPα [127]. The amyloidogenic pathway activity, and by extension Aβ production, is attenuated by cholesterol lowering drugs, metal chelators, steroid hormones or non-steroidal anti-inflammatory drugs [128]. Decreased cellular cholesterol levels disrupt the lipid raft’s structure and function, leading to a concomitant decrease in Aβ and an increase in sAPPα formation [129,130].

The products of β, and γ secretase cleavage are peptides with 39 to 42 amino acids; Aβ40 is the most common and Aβ42 is the most susceptible form to conformational changes leading to amyloid fibrillogenesis. Lower levels of Aβ42 are present in healthy individuals and higher levels in patients with AD (e.g., in familial AD). An increased Aβ42 to Aβ40 ratio has been observed in AD associated mutations in the APP gene [131] and in the PSEN1 gene for preselin1, the catalytic subunit of the γ-secretase complex [132,133,134,135]. Modified Aβ42 peptides enter the cells via endocytosis and lead to lysosomal fusion dysfunction [136]. The overall effect is an enhanced transport of vesicles by the exosomal pathway with increased shedding of modified Aβ peptides into the extracellular space and a reduced Aβ digestion rate by macroautophagy [136]. In AD, Aβ42 oligomers promote neuronal death by:

(a) Reducing Ca^2+^/Calmodulin-dependent protein kinase II synaptic distribution and thus decreasing the density of AMPA synaptic receptors [137];
(b) Uncoupling metabotropic glutamate receptors’ (mGluR5) dependent activation of PKC [138];
(c) Binding with PrP^c^ to the mGluR5 and stimulating mGluR5 clustering at synapses; mGluR5 seems to act as a scaffold for the Aβ oligomer—PrP^c^ signalling complexes [139,140,141,142,143,144,145,146,147];
(d) Binding with PrP^c^ to *N*-methyl-D-aspartate receptors (NMDAR) and
(e) Reducing glutamate reuptake thus promoting an increased NMDAR and mGluR5 mediated entry of Ca^2+^ [148].

PrP^c^ binding to NMDAR enhances its desensitisation and Aβ42 binding attenuates its desensitisation, thus increasing the probability of intracellular Ca^2+^ overload [149,150]. The overall effect of Aβ42 oligomers on the nerve cells is loss of synapses and ultimately cell death due to intracellular Ca^2+^ overload, promoted by increased Ca^2+^ release from intracellular stores and persistent Ca^2+^ entry through NMDARs with attenuated desensitisation.

The modification of Aβ into toxic misfolded Aβ monomers, oligomers and intermediate products [151,152] is accelerated by Aβ oxidation [153]. The oxidation of Aβ42 at the methionine residue 35, Aβ42-MET35-OX, promoted by H_2_O_2_ or copper ions, seems to accelerate the production of toxic Aβ42 products. Also, Aβ42-MET35-OX leads to further protein oxidation and lipid peroxidation and increased oxidative stress [153]. This Aβ42 associated oxidative stress depends on the formation of a sulphur-centred, transient free radical at the methionine residue [153]. In a neuroblastoma cell model, ROS (*i.e.*, H_2_O_2_) catalysed by copper ions promote stabilization of Aβ oligomers by dityrosine cross-links in Aβ42, thus promoting internatilsation of toxic Aβ into lysosomes [154]. *In vitro*, the dityrosine crosslinked Aβ oligomers had the capability to assemble further to form amyloid fibrils. The authors also demonstrated the presence of dityrosine cross-links within plaques in brain samples of patients with AD [154].

Tau is a microtubule-associated protein, located mainly in axons, that stabilises neuronal microtubules during intracellular transport [155]. Tau is necessary for the outgrowth of neuritis-axons or dendrites [156]. In AD, tau is phosphorylated at Ser262 or at Ser214 leading to its detachment from microtubules [157]. AD associated tau is hyperphosphorylated at many sites [158,159,160], often at Ser-Pro or Thr-Pro motifs, and abnormal tau phosphorylation occurs before tau aggregation [161]. The main consequences of tau hyperphosphorylation are the disappearance of microtubules, the breakdown of intracellular traffic and the “dying back” of axons. During AD, tau is redistributed from an axonal to a somato-dendritic pattern which could be the result of an increased tau synthesis as a response to increased tau hyperphosphorylation [161]. Although tau is a very soluble protein it can still form tau aggregates that combine into neurofibrillary tangles (NFT), composed of twisted filaments of hyperphosphorylated tau protein [162]. Oxidation, glycation and proteolytic fragments of tau promote tau aggregation [163,164,165,166]. According to post-mortem reports, NFTs are highly associated with neuronal loss and the severity of cognitive decline [167,168], while Aβ plaques appear at the presymptomatic stage and their levels stabilize relatively early in the disease process [169]. A recent study on five patients with AD and five age-matched healthy controls demonstrated longitudinal changes in tau pathology in patients with AD that was consistent with post-mortem observation of tau pathology in the different stages of AD and correlated with the rate of cognitive decline in patients with AD [170].

### 2.2. Unfolded Protein Response in Alzheimer’s Disease

Despite being one of the most common neurodegenerative disorders, few studies evaluated the impact of the UPR on AD *in vivo*; most of the studies are correlative associations or cell culture experiments [1]. Several studies imply that Aβ interferes with ER functioning. Analyses of AD post-mortem brain revealed altered levels of ER stress markers, like GRP78/BiP, IRE1α, PERK, eIF2α phosphorylation and PDI [4,171,172,173,174]. However, there may be contradictory results when following a single marker. For example, there are reports of no change [175], an increase [176,177] or a decrease [178] in the expression of GRP78/BiP in AD brains. It has been suggested to follow the changes of several factors, for example GRP78/BiP and PDI to assess the UPR activation [4]. Aβ interferes with ER functioning, leading to ER stress in the early stages of AD [177,179,180]. For example, exogenous Aβ induces ER stress in primary neuronal cultures and activates mitochondrial and ER mediated cell death pathways [181,182,183]. In PC12 cells and knock-in mice expressing mutant PS1 it was shown that the up-regulation of protein levels of CHOP/GADD153 was followed by the down-regulation of the anti-apoptotic protein Bcl-2. Concomitantly, the authors observed that mutant PS1 sensitizes cells to the deleterious effects of ER stress culminating in cell death, this effect being attenuated by anti-sense mediated suppression of CHOP/GADD153 production [184]. In the hippocampus of the triple transgenic mouse model of AD (3xTg-AD) increased levels of CHOP/GADD153 precede the increase in BACE and Aβ levels [185]. *Post mortem* immunohistochemical studies of brain tissue samples reveal that markers specific for UPR activation are increased in AD brain tissue compared to non-demented control brain tissue [17,186,187]. Metabolic stress induces phosphorylation of endogenous tau via activation of the UPR. In a cell model, this increase in phosphorylated tau was reversed upon restoration of metabolic homeostasis and was concomitant with the reversal of the levels of UPR markers PERK, IRE1α and Grp78/BiP. The increase in stress induced levels of phosphorylated tau was abolished by the UPR inhibitor TUDCA or by GSK2606414, an inhibitor of the PERK signalling pathway [188]. Tau increased the levels of ubiquitinated proteins in the brain thus leading to activation of the UPR; depletion of soluble tau levels reversed UPR activation [189]. *In vitro* and *in vivo* models of tau over-expression and AD brains imply that soluble tau impairs ERAD and the result of this impairment is the activation of UPR in the ER [189].

**Figure 3:**
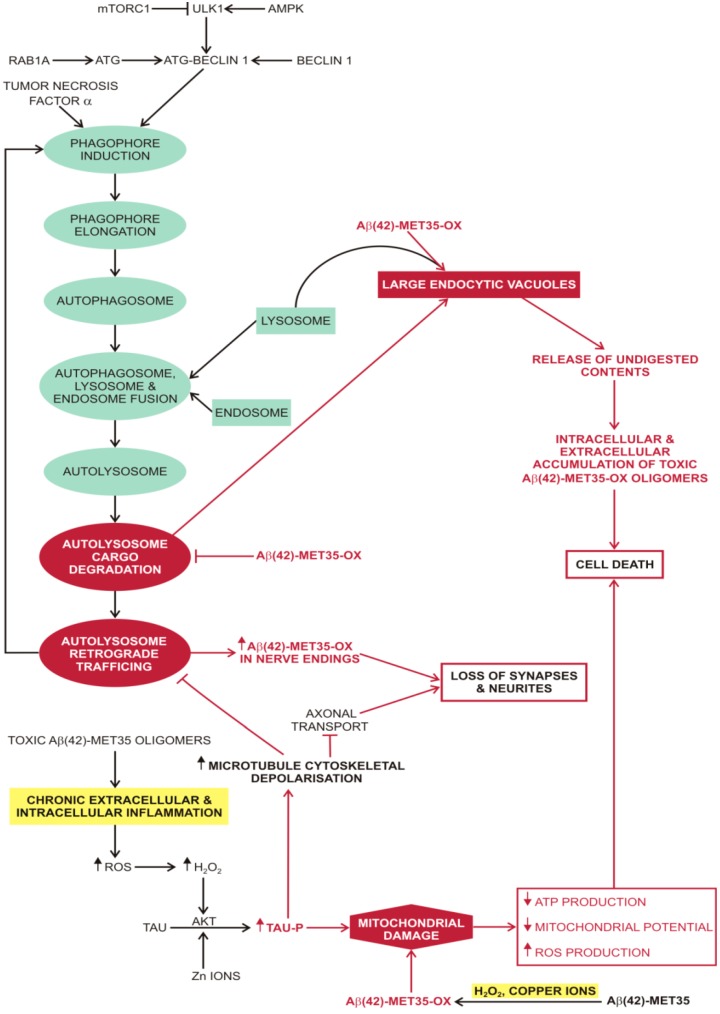
Macroautophagy and oxidated Aβ. Oxidised Aβ peptides attenuate macroautophagy and mitochondrial function. This toxic effect is further exacerbated by the formation of soluble Aβ oligomers that stimulate chronic inflammation with increased production of ROS. A positive feedback loop develops between chronic inflammation and the production of oxidised Aβ peptides, leading to loss of synapses and neurites and ultimately cell death. Abbreviations: ⊥ (attenuation); ↓ (decreased); ↑ (increased); Aβ42-MET35 (soluble amyloid β-peptide with a single methionine residue at position 35); Aβ42-MET35-OX (soluble amyloid β-peptide with a single oxidised methionine residue at position 35); AKT (protein kinase B); AMPK (5’ AMP-activated protein kinase); ATG (autophagy-related protein); ATP (adenosine triphosphate); Copper ions (copper ions in different oxidative states from +1 to +4); mTORC1 (mammalian target of rapamycin complex 1); RAB1A (Ras-related protein Rab-1A); ROS (reactive oxygen species); TAU (tau protein); TAU-P (phosphorylated tau protein); TNFα (tumour necrosis factor alfa/cachexin); ULK1 (serine/threonine-protein kinase).

### 2.3. Macroautophagy in Alzheimer’s disease

Compared to Aβ42, oxidised Aβ42 is more resistant to degradation by autophagy and endosome-lysosome fusion (Figure 3) which further contributes its accumulation and toxic effects due to release of undigested contents into the cytosol [190,191,192,193]. The toxicity of amyloid β-peptide derivatives decreases with protofibril and fibril formation and terminates in the formation of stable and inert amyloid plaques [151,152]. Increased ROS formation, inside or outside the cell, favours the transition of Aβ monomers into toxic forms and also stimulates the breakdown of microtubule cytoskeleton by promoting zinc or H_2_O_2_ induced tau phosphorylation [152,194]. Impaired mitochondrial transport, due to the breakdown of microtubule cytoskeleton, impairs Ca^2+^ homeostasis in synapses and contributes to the loss of synaptic connections in the brain [195]. Aβ42 toxic forms and tau contribute to mitochondrial dysfunction; modified Aβ42 attenuates complex IV (*i.e.*, cytochrome c oxidase) activity and tau attenuates complex I (*i.e.*, NADH-CoQ reductase) activity [195]. In summary, hyperphosphorylated tau, together with toxic forms of Aβ42 lead to an increased production of ROS, a reduced mitochondrial concentration at the synapses, progressive mitochondrial damage with subsequent reduction in ATP production and reduced intracellular Ca^2+^ homeostasis, increased apoptosis signalling, and finally to apoptosis or cell necrosis [195,196]. Not only do toxic β-Amyloid fragments target mitochondria and cause mitochondrial dysfunction [197], but there is some evidence that mitochondrial cytochrome oxidase is defective in AD [198]. AD patients have a significantly decreased cytochrome c oxidase activity in the temporal cortex and hippocampus. These brain regions are involved in processing sensory input, memory and spatial memory [198].

## 3. Parkinson’s Disease

Parkinson’s disease (PD) is the second most common degenerative disorder of the central nervous system, affecting about 1% of people over the age of 60, with diffuse motor and non-motor clinical signs [199]. Animal studies on knock-out mice have linked αSYN with spatial learning and working memory [200]. The primary symptoms of PD are caused by death of dopamine-secreting cells in the *pars compacta* region of the *substantia nigra*. The characteristic pathological changes in the brain are cytosolic inclusions that displace other cell components, *i.e.*, the Lewy bodies (LBs); their number increases with cognitive decline [201]. αSYN is a key component of LBs, other associated proteins are ubiquitin, neurofilament protein, αB crystallin and tau proteins [202,203,204,205,206]. Point mutations in the αSYN gene lead to early onset familial forms of PD with typical PD pathogenesis [207,208,209,210].

### 3.1. α-Synuclein (αSYN)

αSYN is predominantly localised to presynaptic terminals of the central nervous system; it is in a membrane bound and in a predominantly cytosolic form, and modulates vesicular release of dopamine [211,212,213] by attenuating dopamine release under circumstances of repeated firing [214]. αSYN is also localised to the nucleus of mammalian brain neurons [215] and to the inner membrane of neural mitochondria, where it inhibits the complex I activity of the respiratory chain [216]. In addition to modulation of synaptic transmission, αSYN also binds with cytoskeleton associated proteins, for example with tubulin and microtubule associated proteins like tau [217,218]. Therefore, αSYN also modulates the functioning of the neuronal Golgi and vesicle trafficking [219].

*In vivo*, αSYN can be present as a soluble unfolded protein that aggregates into progressively less soluble oligomers, protofibrils and insoluble amyloid fibril form and ultimately LBs [220]. αSYN overexpression is associated with a disruption of the microtubule network [217,221,222,223,224,225,226]. As a general rule, the interaction of αSYN with cytoskeleton associated proteins is stronger for αSYN multimers than for the monomers. For example, in a human neuronal cell line model, a mixture of oligomers and protofibrils of wild type αSYN multimers reduced tau promoted microtubule assembly. In contrast microtubule movement was significantly reduced by αSYN oligomers, but not by fibrils, nor by the mixture of oligomers and protofibrils of the wild type αSYN multimers [8].

Long term imaging *in vivo* demonstrated that the inclusion of preformed αSYN fibrils can seed the progressive conversion of endogenous αSYN into neuronal inclusions that resemble the human LBs and lead to selective neuronal degeneration [227]. This finding supports the suggestion that PD is a neurodegenerative disorder that involves spreading aggregation of specific proteins through the brain by a prion-like mechanism [228,229,230]. Therefore, just an increased level of αSYN is sufficient to promote aggregation and development of PD with LBs [231,232].

### 3.2. Unfolded Protein Response in Parkinson’s Disease

PD is a protein misfolding disorder and chronic ER stress contributes to degeneration of dopaminergic neurons of the *substantia nigra* [1]. ER stress was also identified in neuronal cultures generated from induced pluripotent stem cells from PD patients [233]. Overexpression of ER chaperone GRP78/BiP was neuroprotective in mice [234], in contrast to the removal of ATF6, which enhanced the susceptibility to PD-inducing neurotoxins [235,236]. Salubrinal, an inducer of eIF2α phosphorylation [237] was neuroprotective in αSYN transgenic mice [238]. Combining cellular PD models with biochemical reconstitution assays revealed that αSYN inhibited ATF6 signalling directly through physical interactions and indirectly through restricted incorporation of ATF6 into COPII vesicles. Impaired ATF6 signalling was associated with decreased ERAD function and increased pro-apoptotic signalling [239]. There are very few data on UPR activation from *post*-*mortem* studies on PD patients. However, the immunohistochemical localisation of phosphorylated PERK and eIF2α was characterised in *post*-*mortem* samples of the *substantia nigra* (SN). Immunoreactivity for phosphorylated PERK and eIF2α was observed in PD samples in neuromelanin containing neurons of the SN, while these markers were absent in samples from control subjects [240]. Intracellular accumulation of αSYN is also present in the sporadic neurodegenerative disorder multiple system atrophy (MSA). Double immunohistochemistry using fluorescent antibodies revealed increased phosphorylation of PERK, eIF2α, and IRE1 activity that were closely associated with glial cytoplasmic inclusions containing αSYN [241].

### 3.3. Macroautophagy in Parkinson’s Disease

**Figure 4:**
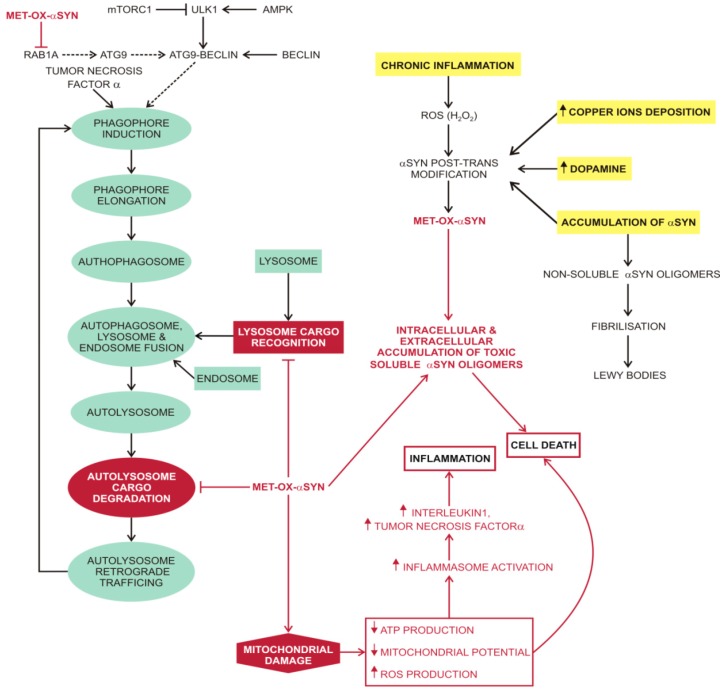
Macroautophagy and oxidised αSYN. Several processes, e.g., chronic inflammation, copper ion deposition, accumulation of dopamine or even just intracellular accumulation of αSYN promote oxidation of αSYN. Oxidised αSYN attenuates macroautophagy and mitochondrial function. This toxic effect is further exacerbated by the formation of soluble and autophagy resistant αSYN oligomers that stimulate chronic inflammation with increased production of ROS. A positive feedback loop develops between chronic inflammation and the production of soluble αSYN oligomers, leading to cell death. Abbreviations: ⊥ (attenuation); ↓ (decreased); ↑ (increased); αSYN (alpha-synuclein); ATG (autophagy-related protein); ATP (adenosine triphosphate production); Copper ions (copper ions in different oxidative states); MET-OX-αSYN (alpha-synuclein oxidised on methionine residues); mTORC1 (mammalian target of rapamycin complex 1); POST-TRANS (post-translational) RAB1A (Ras-related protein Rab-1A); ROS (reactive oxygen species); TNFα (tumour necrosis factor alfa/cachexin); ULK1 (serine/threonine-protein kinase).

The soluble, unfolded αSYN monomer is degraded by CMA [220]. Attenuation of this autophagy pathway could precipitate the development of PD. The most common cause of familial PD are mutations in leucine-rich repeat kinase 2 (LRRK2). LRRK2 is also degraded by the CMA pathway. The most common mutant form of LRRK2, G2019S, is poorly degraded by this pathway and also interferes with organization of the CMA translocation complex. This interference leads to defective CMA degradation of αSYN, and formation of αSYN oligomers at the lysosomal membrane that further block CMA and precipitate the formation of LBs in PD patients with mutant LRRK2 [75]. Attenuation of CMA leads to a compensatory increase in autophagy, an accumulation of autophagosomes, and ultimately to cell death due to the release of undigested contents into the cytosol [242]. αSYN oligomers, protofibrils and amyloid fibrils cannot be digested by autophagy. Posttranslational modifications of αSYN, for example by phosphorylation, ubiquitination, nitration or oxidation, also reduce autophagosome degradation and promote αSYN oligomerisation. The toxic effect of these posttranslational modifications can be compounded by the propensity of αSYN to bind to various molecules in neuronal cells [243]. For example, iron ions, dopamine or H_2_O_2_ oxidise methionine residues in the αSYN monomer, that is the predominant form of oxidised αSYN, and also promote the formation of stable oligomers with a resistance to fibrillization that is proportional to the number of oxidised methionine residues [244]. The oxidised αSYN monomer can interact with lipids, as well as with proteins, changing their redox state and function thus sharing similarities with the Aβ42-MET35-OX [245]. For example, the oxidised αSYN monomer disrupts autophagy (Figure 4) at several stages (inhibits RAB1A activity, attenuates chaperone-mediated autophagy, autophagosome cargo degradation and endolysosome content digestion) and also disrupts mitochondrial function by damage to complex I of the electron transport chain [71,246,247] thus promoting apoptosis or cell necrosis.

Mitochondrial dysfunction, associated with oxidative stress, can trigger αSYN accumulation and aggregation [248]. Recessive forms of familial PD are associated with mutations in proteins pink1 and parkin, that regulate mitophagy, mitochondrial function and biogenesis [249,250,251,252,253,254]. *PINK1* gene encodes a serine/threonine kinase with a mitochondrial targeting sequence and *PARKIN* gene encodes an E3 ubiquitin ligase which forms part of the ubiquitin-proteasome system [255]. It is assumed that both function in the same pathway whereas pink1 is upstream of parkin [255]. Loss of mitochondrial membrane potential due to oxidative stress, mitochondrial DNA mutations or herbicides, e.g., paraquat [256,257], leads to translocation of parkin from cytosol to depolarized mitochondria where it ubiquitinates many outer mitochondrial membrane proteins (e.g., mitofusins and Miros), thus promoting colocalization of mitochondria with autophagy marker LC3 and initiating mithophagy [256]. Binding of pink1 to the translocase of the outer mitochondrial membrane precedes parkin binding and both proteins seem to interact with each other [258,259]. During prolonged mitochondrial depolarization parkin also interacts with autophagy/beclin-1 regulator 1 protein (AMBRA1), initiating AMBRA1 promoted autophagy of depolarised mitochondria by activation of class III phosphatidylinositol 3-kinase complex [260,261]. Mutated pink1 and parkin proteins can lead to accumulation of oxidized lipids, proteins and DNA from damaged mitochondria thus increasing the risk of cell death [262]. However, excessive mitophagy, not balanced with sufficient mitochondrial biogenesis, may over-stress the remaining mitochondria, inducing mitochondrial damage and triggering cell death [263,264,265,266].

## 4. Prion Diseases

Prion diseases (PrDs) are fatal, infectious neurodegenerative disorders that affect humans and other mammals. In humans, prion diseases are acquired, familial, or sporadic. Humans can be infected by contaminated food products or iatrogenic blood transfusion [267,268,269,270,271,272,273,274,275,276,277,278,279,280]. 10%–15% of patients with PrD have a genetic form of the disease, with more than forty different mutations of the human PrP gene, associated with the three heritable forms of prion disease [281]. Creutzfeldt-Jakob disease (CJD) is the most common form of PrD in humans and is characterised by a spongiform degeneration of the brain accompanied by the accumulation of a misfolded and protease-resistant form of the normal form of prion protein (PrP^c^), the infectious prion protein isoform (PrP^Sc^) [282]. PrP^c^ is essential for development of PrDs; replication and release of PrP^Sc^ by implants of prion-infected brain tissue failed to elicit PrD pathology in host PrP knockout brains [283] and transgenic mice with PrP^c^ neuronal depletion during prion infection showed continued extra-neuronal PrP^Sc^ accumulation but without neuronal loss or clinical disease [284]. PrP^c^-dependent neurotoxicity induced by PrP^Sc^ occurs independently of prion replication [146]. Binding of Aβ to membrane-associated PrP^c^ triggers signalling via fyn, and causes hyperphosphorylation of tau, accompanied by synaptic and cognitive impairments [285]. PrDs are caused by the conversion of the host PrP^c^ to the misfolded form of PrP^Sc^ with a β-sheet-rich conformation; the PrP^Sc^ has the ability to convert more PrP^c^ into PrP^Sc^, by propagating its misfolding, resulting in the accumulation of misfolded and aggregated PrP^Sc^ in the brain [282,286]. Prion conversion occurs after PrP maturation [287,288] either at the plasma membrane [289], and/or following endocytosis [287,288,289,290,291,292,293,294]. Whether the conversion of PrP^c^ to PrP^Sc^ requires the presence of a third essential factor, for example the putative protein X, has yet to be proved [295].

Protein aggregates can be seen in other neurodegenerative disorders, for example Aβ and microtubule-associated protein tau in Alzheimer’s disease or αSYN in Parkinson’s disease. Self-propagation of protein aggregates has also been demonstrated in an *in vivo* animal model of PD; the inclusion of preformed αSYN fibrils seeded progressive conversion of endogenous αSYN into neuronal inclusions that resembled the human LBs [227]. Also PrP^c^ and Aβ co-regulate some key cognitive processes, e.g., learning and memory, at the level of synaptic plasticity and neuronal survival [117]. For example, PrP^c^ accentuates NMDA receptor desensitization on nerve cells while Aβ42 attenuates receptor desensitization; PrP^c^ contributes to Aβ42 oligomers mediated nerve cell toxicity [138,139,141,142,143,144,145,146,147].

### 4.1. Normal Form of Prion Protein (PrP^c^)

PrP^c^ is an extracellular protein enriched in α-helix domains, expressed in neurons of the brain and spinal cord, and secured *via* a glycosylphosphatidylinositol (GPI) anchor to the outer surface of the plasma membrane in cholesterol and sphingolipid-rich microdomains [296,297,298]. GPI-anchored PrP^c^ is not a transmembrane protein and cannot transduce signals into the cytosol, but can form signalling complexes with many molecules, for example with stress inducible protein 1 [299,300], neural cell adhesion molecule [301,302] and copper ions [303,304]. Expression levels of PrP^c^ correlate with Cu/Zn superoxide dismutase, glutathione reductase and cytochrome c oxidase activities, copper-concentration and severity of hypoxia suggesting that PrP^c^ has a protective role against reactive oxygen species (ROS), for review see [305]. Failure of PrP^c^ to buffer increased intracellular copper ion concentrations leads to mitochondrial dysfunction, due to a reduced cytochrome c oxidase activity, and to activation of mitochondria mediated apoptosis [306]. It has been suggested that copper ion imbalance is an early change in prion disease [307]. PrP^c^ signalling complexes activate the cAMP-dependent protein kinase A and ERK1/2 pathways, thus influencing neural survival, neuritogenesis, and memory formation [308,309].

Lateral movement to detergent-soluble plasma membrane domains enables PrP^c^ internalisation via endosomes and contact with the autophagy degradation machinery [310]. In physiological conditions, most of the internalised PrP^c^ is recycled back to the plasma membrane [311]. Additional sources of cytosolic PrP^c^ are due to (a) retro-translocation from the ER [312,313]; (b) avoidance of translocation into the ER due to inefficiencies of the signal sequence [314,315,316]; or (c) ER pre-emptive quality control preventing translocation of newly formed PrP^c^ molecules during ER stress [317]. Cytosolic PrP^c^ is normally present in low concentrations due to rapid degradation; inefficient degradation leads to pathologic cytosol accumulation and formation of PrD promoting aggregates [281]. Cytosolic PrP^c^ protects human primary neurons from Bax-mediated apoptosis [318,319,320]. Co-expressed with PrP^c^, but with a completely different amino acid sequence and unknown function, is the alternative prion protein form that localizes to mitochondria and is up-regulated by ER stress [321].

Although the physiological role of mammalian prion protein (PrP^c^) is not fully understood, there is some evidence suggesting that it could play an important role as an antioxidant, an endogenous scavenger, protecting other structural and signalling proteins, since it has a high number of methionine residues [322,323,324]. Oxidation of these residues, usually during copper redox cycling, transforms the hydrophobic thioether side chain of methionine into a hydrophilic sulfoxide and can also affect the structural integrity of proteins [325]. PrP^c^ could also play an active role since methionine sulfoxidation is involved in cell signalling [326]. In healthy tissue the posttranslational modification of methionine is reversed by cellular methionine sulfoxide reductases [327]. The postulated age related degradation of this reversible oxidation could lead to progressive accumulation of oxidised methionine residues on the PrP^c^ and contribute to protein misfolding by initiating first the transition to a monomeric molten globule form with a conserved helical content, second promoting extended β-strand structures that lack a cooperative field and finally the appearance of the infective, self-propagating, autocatalytic, misfolded isoform PrP^Sc^ [328,329]. The transition of PrP^c^ *N*-terminal region from a random coil to a β-sheet structure, predominant over the α-helices content, transforms the soluble and protease-sensitive PrP^c^ into the oxidised, insoluble and relatively more protease-resistant PrP^Sc^ [330,331].

**Figure 5:**
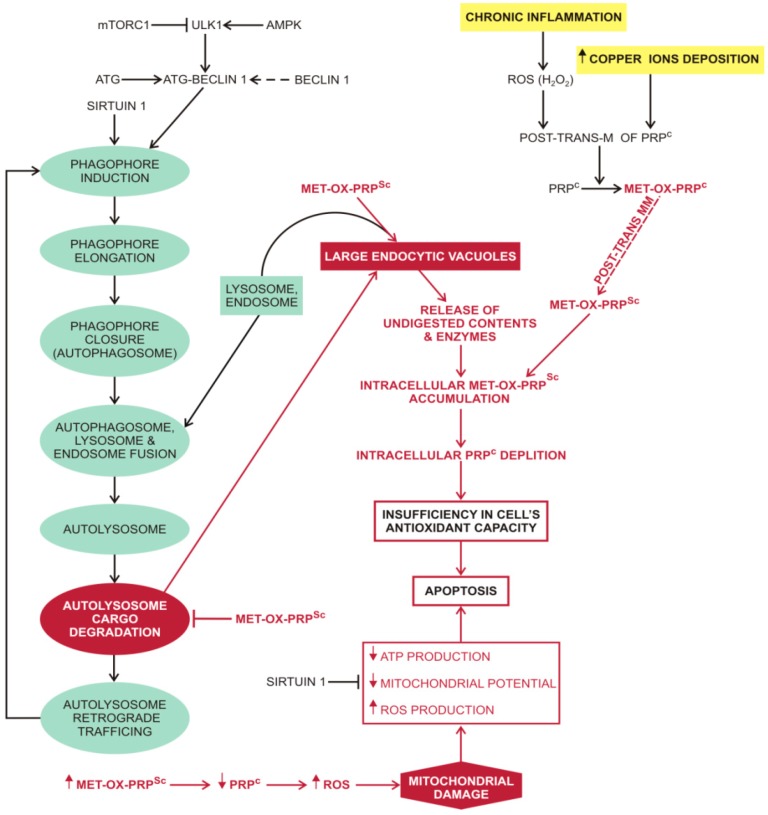
Macroautophagy of oxidated PrP^Sc^. The predisposing factors for the transformation of the native prion protein (PrP^c^) into the infectious, self-propagating prion protein form (PrP^Sc^) are chronic brain inflammation and copper ion deposition. They promote many post translational modifications of PrP^c^, including oxidation of its numerous methionine residues, to the prion protein in molten globule form (met-ox-PrP^c^). Further post translational modifications of met-ox-PrP^c^ can also include a transformation into an oxidised and self-propagating infectious isoform of prion protein (met-ox-PrP^Sc^). The met-ox-PrP^Sc^ is resistant to autophagy; attenuates autolysosome cargo degradation and also promotes the formation of large endocytic vacuoles that tend to release their undigested contents, including enzymes, into the cytosol. An intracellular increase in met-ox-PrP^Sc^, due to its resistance to removal by autophagy and propensity for self-propagation, leads to an intracellular reduction of PrP^c^. The intracellular reduction of PrP^c^ changes the cell’s global oxidative-redox balance which is reflected in mitochondrial damage, a contributing factor to the met-ox-PrP^Sc^ induced apoptosis. Although chronic brain inflammation seems to be important for initiating the process of PrP^Sc^ production, it is not necessary to sustain it, since the PrP^Sc^ only needs the PrP^c^ molecules for its propagation. Abbreviations: ⊥ (attenuation); ↓ (decreased); ↑ (increased); AMPK (5′ AMP-activated protein kinase); ATG (autophagy-related protein); ATP (adenosine triphosphate); Copper ions (copper ions in different oxidative states from +1 to +4); MET-OX-PRP^c^ (oxidised prion protein in molten globule form); MET-OX-PRP^Sc^ (oxidised and self-propagating prion protein); mTORC1 (mammalian target of rapamycin complex 1); POST-TRANS-M (post-translational modification); POST-TRANS-MM (post-translational modifications); PrP^c^ (normal form of prion protein); PrP^Sc^ (infectious isoform of prion protein); ROS (reactive oxygen species); SIRT1 (NAD-dependent deacetylase sirtuin-1); ULK1 (serine/threonine-protein kinase).

PrP^Sc^ monomers, small oligomers and oligomers induce nerve cell death after internalization and accumulation in the endolysosomal compartment where they cause lysosomal damage with subsequent proteolytic enzyme leakage and activation of caspase-dependent apoptosis [332,333]. Autophagy efficiency is also attenuated (Figure 5), due to PrP^Sc^ protease-resistance; aggregation of misfolded PrP^Sc^ also leads to mitochondrial failure [334,335,336]. The prion fibrils seem to be a stable and inert form of the prion protein [337]. In contrast to Parkinson’s and Alzheimer’s disease, inflammation is less pronounced in prion disease, suggesting that apoptosis predominates over cell necrosis [338]. Assuming that the primary role of PrP^c^ is to act as a global cell antioxidant, regulating the oxidative state of structural and signalling proteins, the conversion of the soluble PrP^c^ proteins to less soluble and aggregation prone oxidised PrP^Sc^ proteins could lead to a major depletion of PrP^c^ molecules and a catastrophic disruption in the cell’s global oxidative-redox balance, thus leading to apoptosis.

### 4.2. Unfolded Protein Response in Prion Diseases

ER stress has been described in models of infectious forms of PrDs [339,340]. However its contribution to pathogenesis is not clear, as disease progression does not seem to be affected by deficiency of *Xbp1* or caspase 12 [341,342]. When interpreting the role of caspase-12 in PrDs one should consider that caspase-12 is also involved in regulating inflammatory cytokines in mice [343] and that in the great majority of the human population caspase-12 is expressed as a truncated, inactive protein [344].

It was also observed that prion replication led to sustained eIF2α phosphorylation responsible for the neurological and behavioural impairment of the prion-diseased infected mouse model through repression of synaptic proteins that are synthesized through the ER-Golgi secretory pathway [27]. The detrimental effects of sustained eIF2α phosphorylation could be reversed by over-expression of GADD34 or by lentivirally-mediated anti-PrP RNAi; salubrinal accentuates these effects by inhibiting eIF2α-P dephosphorylation [27]. Oral administration of a PERK inhibitor also protected animals from PrD related neurodegeneration [345]. However, pharmacological induction of ER stress with DTT or thapsigargin, in neuronal cell lines that expressed mutant PrP, decreased PrP^c^ aggregation and PrP^Sc^ accumulation [346]. One explanation that could reconcile the divergent results of prion research is that UPR activation is beneficial in prevention of and in the early stages of PrDs, however with progressive accumulation of misfolded PrP^c^ and PrP^Sc^, derangements of UPR develop that contribute to progression of the disease. ER stress markers in the brain are upregulated in prion infected mice and in *post*-*mortem* samples of patients with CJD but to a considerably lesser degree than in the brain samples of AD patients [181]. An immunohistochemical study looking at the localization of phosphorylated PERK and eIF2α-P in 25 human patients could not confirm the involvement of UPR in human prion diseases [181]. Only human prion disease cases that showed concomitant AD pathology had increased presence of pPERK and p-eIF2α, suggesting that these markers were not related to the prion pathology [17]. Therefore comorbidity of neuropathological processes is an important factor in the study of UPR activation in human neuropathology and the precise role of PrDs in modulating the UPR needs further examination [17].

### 4.3. Macroautophagy in Prion Diseases

Autophagy vacuoles were described in neurons in animal experimental models of PrD [347,348] and also in prion-infected neuronal cell cultures [349]. The location of autophagy vacuoles was in neuronal perikarya, neurites and synapses in experimentally induced scrapie, Creutzfeldt-Jakob disease (CJD) and in Gerstmann-Sträussler-Scheinker (GSS) syndrome [350,351]. The presence of autophagy vacuoles was associated with an up-regulation of *scrapie responsive gene 1* in animal models of PrDs and in brains of patients with sporadic CJD [352,353,354,355,356]. PrP^c^ modulation of autophagy was demonstrated by an increased expression of LC3-II in neurons of Zürich I *Prnp*−*/*− mice; this was attenuated by the reintroduction of an octapeptide region rich PrP^c^ into these cells [357]. It has been suggested that PrD modulated autophagy precipitates apoptosis, thus contributing to the formation of spongiform changes in the brain [350,358,359]. It is also possible that, before PrD related changes of PrP^c^ and during the early stages of PrD, autophagy contributes to degradation of PrP^c^ and PrP^Sc^. Thus the failure of autophagy leads to activation of apoptosis and progression to PrD. The latter explanation is consistent with the observation of reduced levels of lysosomal activation marker LAMP-2 in the brain tissue of prion-infected galactin-3–/– mice, prion-infected wild-type and in galactin-3–/– mice where lower mRNA levels of autophagy markers Beclin-1 and Atg5 were detected, compared to mock infected control brains. This finding supports the hypothesis that endosomal/lysosomal dysfunction and reduced autophagy may contribute to the development of PrD [360]. Also the drug imatinib enhances lysosomal degradation of PrP^Sc^ [361], stimulates autophagy and/or autophagosome formation [362] and even delays, but does not prevent, neuroinvasion of PrP^Sc^ and the onset of clinical disease in prion-infected mice [363]. In summary, reduction of cellular of PrP^c^ and PrP^Sc^ can be achieved by pharmacological stimulation of autophagy in an mTOR-independent manner (e.g., with lithium, trehalose, imatinib) or by inhibition of mTOR (e.g., with rapamycin). Compared to mock-treated control mice, rapamycin and lithium prolonged prion incubation times in contrast to trehalose and imatinid that delayed peripheral accumulation of PrP^Sc^ [5,92,93,364,365,366,367]. Inhibition of autophagy by pharmacological interference and siRNA gene silencing attenuated autophagy-mediated reduction of cellular PrP^Sc^ [5]. It seems that the further development of autophagy inducing compounds could lead to effective treatment of PrDs.

PrP^Sc^ is found in many different cell locations: on the plasma membrane, in the endolysosomal system, in the endosomal recycling compartment, in the trans Golgi network and Golgi, in the autophagic pathway and in the cytosol [290,291,293,294,368,369,370]. Autophagy is the major route of PrP^Sc^ degradation [371], alternative degradation pathways for newly endocytosed PrP^Sc^ are through the endolysosomal pathway [294] and the Golgi QC pathway [372,373]. Proteasome inhibition increased PrP^Sc^ levels in chronically-infected cultured cells and this increase was not accompanied by increased PrP^c^ expression [373]. In contrast, PrP^Sc^ is not ubiquitinated for degradation by the proteasome to a significant degree, suggesting proteasomal PrP^Sc^ degradation by a non-ubiquitin pathway, for review see [374]. Formation of insoluble PrP^Sc^ forms (*i.e.*, aggresomes) could be facilitated under conditions of increased cell stress that leads to proteasome inhibition or in the later stages of prion disease [375,376]. Recently, a comprehensive synthesis of prion formation, trafficking and degradation pathways has been presented [374]. PrP^Sc^ is assumed to form at the plasma membrane, in endosomes, in recycling vesicles or in lysosomes. PrP^Sc^ recycling to the plasma membrane stimulates prion propagation. The intracellular transport of PrP^Sc^ follows three pathways. One pathway enables PrP^Sc^ recycling to the plasma membrane, the other two target PrP^Sc^ for degradation. Newly formed and mature forms of PrP^Sc^ are degraded by different pathways. Newly formed PrP^Sc^ is moved by retrograde transport to the trans Golgi network (TGN) and Golgi, subjected to Golgi quality control and sent to lysosomes for degradation. Mature PrP^Sc^ forms, representing the majority of total cellular PrP^Sc^, are moved to lysosomes via the endolysosomal, autophagy and proteasome degradation pathways [5,93,371,373]. To summarise, the increased cytosolic presence of PrP^Sc^ is due to (a) the lysosomal rupture caused by aggregated PrP^Sc^ that de-stabilise the lysosomal membrane [377]; (b) the low rate of ERAD ubiquitination of PrP^Sc^ for proteasomal degradation in the cytosol [378,379]; and (c) the low degradation rate or failure of the PrP^Sc^ ubiquitin-independent degradation by the proteasome. Thus, PrP^Sc^ accumulates and forms smaller and larger insoluble forms (*i.e.*, aggresomes) that are degraded by macroautophagy.

## 5. Conclusions

Alzheimer’s disease, Parkinson’s disease and prion diseases have different clinical manifestations of neurodegeneration, however, all three share the process of accumulating misfolded, pathological proteins. Proteostasis in these neurodegenerative disorders is further perturbed by age-related brain changes and associated chronic diseases, like for example diabetes mellitus. While both the unfolded protein response and autophagy have an important role in the prevention and attenuation of Alzheimer’s and Parkinson’s diseases progression, only macroautophagy seems to play an important role in the development of prion diseases. Autophagy and unfolded protein response can be modulated by pharmacological interventions. Further research is necessary to better understand the regulatory pathways of both processes, in health and neurodegeneration, to be able to develop new therapeutic interventions.

## References

[1] Hetz C., Mollereau B. (2014). Disturbance of endoplasmic reticulum proteostasis in neurodegenerative diseases. Nat. Rev. Neurosci..

[2] Brown M.K., Naidoo N. (2012). The endoplasmic reticulum stress response in aging and age-related diseases. Front. Physiol..

[3] Lipinski M.M., Zheng B., Lu T., Yan Z., Py B.F., Ng A., Xavier R.J., Li C., Yankner B.A., Scherzer C.R. (2010). Genome-wide analysis reveals mechanisms modulating autophagy in normal brain aging and in Alzheimer’s disease. Proc. Natl. Acad. Sci. USA.

[4] Roussel B.D., Kruppa A.J., Miranda E., Crowther D.C., Lomas D.A., Marciniak S.J. (2013). Endoplasmic reticulum dysfunction in neurological disease. Lancet Neurol..

[5] Heiseke A., Aguib Y., Schatzl H.M. (2010). Autophagy, prion infection and their mutual interactions. Curr. Issues Mol. Biol..

[6] Ravikumar B., Sarkar S., Davies J.E., Futter M., Garcia-Arencibia M., Green-Thompson Z.W., Jimenez-Sanchez M., Korolchuk V.I., Lichtenberg M., Luo S. (2010). Regulation of mammalian autophagy in physiology and pathophysiology. Physiol. Rev..

[7] Szul T., Sztul E. (2011). COPII and COPI traffic at the ER-Golgi interface. Physiology.

[8] Prots I., Veber V., Brey S., Campioni S., Buder K., Riek R., Böhm K.J., Winner B. (2013). α-Synuclein oligomers impair neuronal microtubule-kinesin interplay. J. Biol. Chem..

[9] Spires-Jones T.L., Hyman B.T. (2014). The intersection of amyloid beta and tau at synapses in Alzheimer’s disease. Neuron.

[10] Marchi S., Patergnani S., Pinton P. (2014). The endoplasmic reticulum-mitochondria connection: One touch, multiple functions. Biochim. Biophys. Acta.

[11] Verfaillie T., Rubio N., Garg A.D., Bultynck G., Rizzuto R., Decuypere J.P., Piette J., Linehan C., Gupta S., Samali A., Agostinis P. (2012). PERK is required at the ER–mitochondrial contact sites to convey apoptosis after ROS-based ER stress. Cell Death Differ..

[12] Hedskog L., Pinho C.M., Filadi R., Rönnbäck A., Hertwig L., Wiehager B., Larssen P., Gellhaar S., Sandebring A., Westerlund M. (2013). Modulation of the endoplasmic reticulum-mitochondria interface in Alzheimer’s disease and related models. Proc. Natl. Acad. Sci. USA.

[13] Zampese E., Fasolato C., Kipanyula M.J., Bortolozzi M., Pozzan T., Pizzo P. (2011). Presenilin 2 modulates endoplasmic reticulum (ER)-mitochondria interactions and Ca^2+^ cross-talk. Proc. Natl. Acad. Sci. USA.

[14] Boehning D., Patterson R.L., Sedaghat L., Glebova N.O., Kurosaki T., Snyder S.H. (2003). Cytochrome c binds to inositol (1,4,5) trisphosphate receptors, amplifying calcium-dependent apoptosis. Nat. Cell Biol..

[15] Boehning D., Patterson R.L., Snyder S.H. (2004). Apoptosis and calcium: New roles for cytochrome c and inositol 1,4,5-trisphosphate. Cell Cycle.

[16] Boehning D., van Rossum D.B., Patterson R.L., Snyder S.H. (2005). A peptide inhibitor of cytochrome c/inositol 1,4,5-trisphosphate receptor binding blocks intrinsic and extrinsic cell death pathways. Proc. Natl. Acad. Sci. USA.

[17] Scheper W., Hoozemans J.J. (2015). The unfolded protein response in neurodegenerative diseases: A neuropathological perspective. Acta Neuropathol..

[18] Wang S., Kaufman R.J. (2012). The impact of the unfolded protein response on human disease. J. Cell Biol..

[19] Cao S.S., Kaufman R.J. (2012). Unfolded protein response. Curr. Biol..

[20] Sovolyova N., Healy S., Samali A., Logue S.E. (2014). Stressed to death—Mechanisms of ER stress-induced cell death. Biol. Chem..

[21] Chen Y., Brandizzi F. (2013). IRE1: ER stress sensor and cell fate executor. Trends Cell Biol..

[22] Oikawa D., Kitamura A., Kinjo M., Iwawaki T. (2012). Direct association of unfolded proteins with mammalian ER stress sensor, IRE1β. PLoS ONE.

[23] Thuerauf D.J., Marcinko M., Belmont P.J., Glembotski C.C. (2007). Effects of the isoform-specific characteristics of ATF6 alpha and ATF6 beta on endoplasmic reticulum stress response gene expression and cell viability. J. Biol. Chem..

[24] Deldicque L. (2013). Endoplasmic reticulum stress in human skeletal muscle: Any contribution to sarcopenia?. Front. Physiol..

[25] Dufey E., Sepúlveda D., Rojas-Rivera D., Hetz C. (2014). Cellular mechanisms of endoplasmic reticulum stress signaling in health and disease. 1. An overview. Am. J. Physiol. Cell Physiol..

[26] Brewer G.J. (2014). Alzheimer’s disease causation by copper toxicity and treatment with zinc. Front. Aging Neurosci..

[27] Vannuvel K., Renard P., Raes M., Arnould T. (2013). Functional andmorphological impact of ER stress on mitochondria. J. Cell. Physiol..

[28] Harding H.P., Zhang Y., Ron D. (1999). Protein translation and folding are coupled by an endoplasmic-reticulum-resident kinase. Nature.

[29] Ameri K., Harris A.L. (2008). Activating transcription factor 4. Int. J. Biochem. Cell Biol..

[30] Hetz C. (2012). The unfolded protein response: Controlling cell fate decisions under ER stress and beyond. Nat. Rev. Mol. Cell Biol..

[31] Pereira C. (2013). Crosstalk between endoplasmic reticulum stress and protein misfolding in neurodegenerative diseases. ISRN Cell Biol..

[32] Schröder M., Kaufman R.J. (2005). The mammalian unfolded protein response. Annu. Rev. Biochem..

[33] Averous J., Bruhat A., Jousse C., Carraro V., Thiel G., Fafournoux P. (2004). Induction of CHOP expression by amino acid limitation requires both ATF4 expression and ATF2 phosphorylation. J. Biol. Chem..

[34] Nguyên D.T., Kebache S., Fazel A., Wong H.N., Jenna S., Emadali A., Lee E.H., Bergeron J.J., Kaufman R.J., Larose L. (2004). Nck-dependent activation of extracellular signal-regulated kinase-1 and regulation of cell survival during endoplasmic reticulum stress. Mol. Biol. Cell.

[35] Hu P., Han Z., Couvillon A.D., Kaufman R.J., Exton J.H. (2006). Autocrine tumor necrosis factor alpha links endoplasmic reticulum stress to the membrane death receptor pathway through IRE1alpha-mediated NF-kappaB activation and down-regulation of TRAF2 expression. Mol. Cell Biol..

[36] Simmen T., Lynes E.M., Gesson K., Thomas G. (2010). Oxidative protein folding in the endoplasmic reticulum: Tight links to the mitochondria-associated membrane (MAM). Biochim. Biophys. Acta.

[37] Satpute-Krishnan P., Ajinkya M., Bhat S., Itakura E., Hegde R.S., Lippincott-Schwartz J. (2014). ER stress-induced clearance of misfolded GPI-anchored proteins via the secretory pathway. Cell.

[38] Couve A., Hetz C. (2014). RESETing ER proteostasis: Selective stress pathway hidden in the secretory route. EMBO J..

[39] Reid D.W., Chen Q., Tay A.S., Shenolikar S., Nicchitta C.V. (2014). The unfolded protein response triggers selective mRNA release from the endoplasmic reticulum. Cell.

[40] Koivunen P., Horelli-Kuitunen N., Helaakoski T., Karvonen P., Jaakkola M., Palotie A., Kivirikko K.I. (1997). Structures of the human gene for the protein disulfide isomerase-related polypeptide ERp60 and a processed gene and assignment of these genes to 15q15 and 1q21. Genomics.

[41] Higa A., Chevet E. (2012). Redox signaling loops in the unfolded protein response. Cell Signal..

[42] Cao S.S., Kaufman R.J. (2014). Endoplasmic reticulum stress and oxidative stress in cell fate decision and human disease. Antioxid. Redox Signal..

[43] Olzmann J.A., Kopito R.R., Christianson J.C. (2013). The mammalian endoplasmic reticulum-associated degradation system. Cold Spring Harb. Perspect. Biol..

[44] Appenzeller-Herzog C., Ellgaard L. (2008). The human PDI family: Versatility packed into a single fold. Biochim. Biophys. Acta.

[45] Benham A.M. (2012). The protein disulfide isomerase family: Key players in health and disease. Antioxid. Redox Signal..

[46] Braakman I., Bulleid N.J. (2011). Protein folding and modification in the mammalian endoplasmic reticulum. Annu. Rev. Biochem..

[47] Pollock S., Kozlov G., Pelletier M.F., Trempe J.F., Jansen G., Sitnikov D., Bergeron J.J., Gehring K., Ekiel I., Thomas D.Y. (2004). Specific interaction of ERp57 and calnexin determined by NMR spectroscopy and an ER two-hybrid system. EMBO J..

[48] Dong M., Bridges J.P., Apsley K., Xu Y., Weaver T.E. (2008). ERdj4 and ERdj5 are required for endoplasmic reticulum-associated protein degradation of misfolded surfactant protein C. Mol. Biol. Cell.

[49] Ushioda R., Hoseki J., Araki K., Jansen G., Thomas D.Y., Nagata K. (2008). ERdj5 is required as a disulfide reductase for degradation of misfolded proteins in the ER. Science.

[50] Tavender T.J., Bulleid N.J. (2010). Peroxiredoxin IV protects cells from oxidative stress by removing H_2_O_2_ produced during disulphide formation. J. Cell Sci..

[51] Ramming T., Appenzeller-Herzog C. (2012). The physiological functions of mammalian endoplasmic oxidoreductin 1: On disulfides and more. Antioxid. Redox Signal..

[52] Hwang C., Sinskey A.J., Lodish H.F. (1992). Oxidized redox state of glutathione in the endoplasmic reticulum. Science.

[53] Görlach A., Klappa P., Kietzmann T. (2006). The endoplasmic reticulum: Folding, calcium homeostasis, signaling, and redox control. Antioxid. Redox Signal..

[54] Valko M., Jomova K., Rhodes C.J., Kuča K., Musílek K. (2015). Redox- and non-redox-metal-induced formation of free radicals and their role in human disease. Arch. Toxicol..

[55] Molinari M., Calanca V., Galli C., Lucca P., Paganetti P. (2003). Role of EDEM in the release of misfolded glycoproteins from the calnexin cycle. Science.

[56] Bernales S., McDonald K.L., Walter P. (2006). Autophagy counterbalances endoplasmic reticulum expansion during the unfolded protein response. PLoS Biol..

[57] Ogata M., Hino S., Saito A., Morikawa K., Kondo S., Kanemoto S., Murakami T., Taniguchi M., Tanii I., Yoshinaga K. (2006). Autophagy is activated for cell survival after endoplasmic reticulum stress. Mol. Cell Biol..

[58] Nijholt D.A., de Graaf T.R., van Haastert E.S., Oliveira A.O., Berkers C.R., Zwart R., Ovaa H., Baas F., Hoozemans J.J., Scheper W. (2011). Endoplasmic reticulum stress activates autophagy but not the proteasome in neuronal cells: Implications for Alzheimer’s disease. Cell Death Differ..

[59] Elfrink H.L., Zwart R., Baas F., Scheper W. (2013). Inhibition of endoplasmic reticulum associated degradation reduces endoplasmic reticulum stress and alters lysosomal morphology and distribution. Mol. Cells.

[60] Kaniuk N.A., Kiraly M., Bates H., Vranic M., Volchuk A., Brumell J.H. (2007). Ubiquitinated-protein aggregates form in pancreatic beta-cells during diabetes-induced oxidative stress and are regulated by autophagy. Diabetes.

[61] Helenius A. (1994). How *N*-linked oligosaccharides affect glycoprotein folding in the endoplasmic reticulum. Mol. Biol. Cell.

[62] Groisman B., Shenkman M., Ron E., Lederkremer G.Z. (2011). Mannose Trimming Is Required for Delivery of a Glycoprotein from EDEM1 to XTP3-B and to Late Endoplasmic Reticulum-associated Degradation Steps. J. Biol. Chem..

[63] Lederkremer G.Z., Glickman M.H. (2005). A window of opportunity: Timing protein degradation by trimming of sugars and ubiquitins. Trends Biochem. Sci..

[64] Araki K., Nagata K. (2011). Protein Folding and Quality Control in the ER. Cold Spring Harb. Perspect. Biol..

[65] Hagiwara M., Maegawa K., Suzuki M., Ushioda R., Araki K., Matsumoto Y., Hoseki J., Nagata K., Inaba K. (2011). Structural basis of an ERAD pathway mediated by the ER-resident protein disulfide reductase ERdj5. Mol. Cell.

[66] Hosokawa N., Kamiya Y., Kamiya D., Kato K., Nagata K. (2009). Human OS-9, a lectin required for glycoprotein endoplasmic reticulum-associated degradation, recognizes mannose-trimmed *N*-glycans. J. Biol. Chem..

[67] Bernasconi R., Galli C., Calanca V., Nakajima T., Molinari M. (2010). Stringent requirement for HRD1, SEL1L, and OS-9/XTP3-B for disposal of ERAD-LS substrates. J. Cell Biol..

[68] Riemer J., Hansen H.G., Appenzeller-Herzog C., Johansson L., Ellgaard L. (2011). Identification of the PDIfamily member ERp90 as an interaction partner of ERFAD. PLoS ONE.

[69] Ushioda R., Hoseki J., Nagata K. (2013). Glycosylation-independent ERAD pathway serves as a backup system under ER stress. Mol. Biol. Cell.

[70] Ron E., Shenkman M., Groisman B., Izenshtein Y., Leitman J., Lederkremer G.Z. (2011). Bypass of glycan-dependent glycoprotein delivery to ERAD by up-regulated EDEM1. Mol. Biol. Cell.

[71] Lynch-Day M.A., Mao K., Wang K., Zhao M., Klionsky D.J. (2012). The role of autophagy in Parkinson’s disease. Cold Spring Harb. Perspect. Med..

[72] Klionsky D.J. (2005). The molecular machinery of autophagy: Unanswered questions. J. Cell Sci..

[73] Kabeya Y., Mizushima N., Ueno T., Yamamoto A., Kirisako T., Noda T., Kominami E., Ohsumi Y., Yoshimori T. (2000). LC3, a mammalian homologue of yeast Apg8p, is localized in autophagosome membranes after processing. EMBO J..

[74] Kunz J.B., Schwarz H., Mayer A. (2004). Determination of four sequential stages during microautophagy *in vitro*. J. Biol. Chem..

[75] Orenstein S.J., Kuo S.H., Tasset I., Arias E., Koga H., Fernandez-Carasa I., Cortes E., Honig L.S., Dauer W., Consiglio A. (2013). Interplay of LRRK2 with chaperone-mediated autophagy. Nat. Neurosci..

[76] Majeski A.E., Dice J.F. (2004). Mechanisms of chaperone-mediated autophagy. Int. J. Biochem. Cell Biol..

[77] Kaushik S., Cuervo A.M. (2012). Chaperone-mediated autophagy: A unique way to enter the lysosome world. Trends Cell Biol..

[78] Klionsky D.J., Cuervo A.M., Dunn W.A., Levine B., van der Klei I., Seglen P.O. (2007). How shall I eat thee?. Autophagy.

[79] Shintani T., Huang W.-P., Stromhaug P.E., Klionsky D.J. (2002). Mechanism of cargo selection in the cytoplasm to vacuole targeting pathway. Dev. Cell.

[80] Kanki T., Wang K., Cao Y., Baba M., Klionsky D.J. (2009). Atg32 is a mitochondrial protein that confers selectivity during mitophagy. Dev. Cell.

[81] Lemasters J.J. (2005). Selective mitochondrial autophagy, or mitophagy, as a targeted defense against oxidative stress, mitochondrial dysfunction, and aging. Rejuvenation Res..

[82] Hutchins M.U., Klionsky D.J. (2001). Vacuolar localization of oligomeric a-mannosidase requires the cytoplasm to vacuole targeting and autophagy pathway components in Saccharomyces cerevisiae. J. Biol. Chem..

[83] Klionsky D.J., Cueva R., Yaver D.S. (1992). Aminopeptidase I of Saccharomyces cerevisiae is localized to the vacuole independent of the secretory pathway. J. Cell Biol..

[84] Yuga M., Gomi K., Klionsky D.J., Shintani T. (2011). Aspartyl aminopeptidase is imported from the cytoplasm to the vacuole by selective autophagy in Saccharomyces cerevisiae. J. Biol. Chem..

[85] Mizushima N. (2004). Methods for monitoring autophagy. Int. J. Biochem. Cell Biol..

[86] Nixon R.A., Wegiel J., Kumar A., Yu W.H., Peterhoff C., Cataldo A., Cuervo A.M. (2005). Extensive involvement of autophagy in Alzheimer disease: An immunoelectron microscopy study. J. Neuropathol. Exp. Neurol..

[87] Ghavami S., Shojaei S., Yeganeh B., Ande S.R., Jangamreddy J.R., Mehrpour M., Christoffersson J., Chaabane W., Moghadam A.R., Kashani H.H. (2014). Autophagy and apoptosis dysfunction in neurodegenerative disorders. Prog. Neurobiol..

[88] Petersen A., Larsen K.E., Behr G.G., Romero N., Przedborski S., Brundin P., Sulzer D. (2001). Expanded CAG repeats in exon 1 of the Huntington’s disease gene stimulate dopamine-mediated striatal neuron autophagy and degeneration. Hum. Mol. Genet..

[89] Yu W.H., Cuervo A.M., Kumar A., Peterhoff C.M., Schmidt S.D., Lee J.H., Mohan P.S., Mercken M., Farmery M.R., Tjernberg L.O. (2005). Macroautophagy—A novel Beta-amyloid peptide-generating pathway activated in Alzheimer’s disease. J. Cell Biol..

[90] Webb J.L., Ravikumar B., Atkins J., Skepper J.N., Rubinsztein D.C. (2003). Alpha-Synuclein is degraded by both autophagy and the proteasome. J. Biol. Chem..

[91] Sarkar S., Davies J.E., Huang Z., Tunnacliffe A., Rubinsztein D.C. (2007). Trehalose, a novel mTORindependent autophagy enhancer, accelerates the clearance of mutant huntingtin and alpha-synuclein. J. Biol. Chem..

[92] Aguib Y., Heiseke A., Gilch S., Riemer C., Baier M., Schatzl H.M., Ertmer A. (2009). Autophagy induction by trehalose counteracts cellular prion infection. Autophagy.

[93] Heiseke A., Aguib Y., Riemer C., Baier M., Schatzl H.M. (2009). Lithium induces clearance of protease resistant prion protein in prion-infected cells by induction of autophagy. J. Neurochem..

[94] Schneider J.A., Arvanitakis Z., Bang W., Bennett D.A. (2007). Mixed brain pathologies account for most dementia cases in communitydwelling older persons. Neurology.

[95] James B.D., Bennett D.A., Boyle P.A., Leurgans S., Schneider J.A. (2012). Dementia from Alzheimer disease and mixed pathologies in the oldest old. JAMA.

[96] Davidson Y.S., Raby S., Foulds P.G., Robinson A., Thompson J.C., Sikkink S., Yusuf I., Amin H., DuPlessis D., Troakes C. (2011). TDP-43 pathological changes in early onset familial and sporadic Alzheimer’s disease, late onset Alzheimer’s disease and Down’s syndrome: Association with age, hippocampal sclerosis and clinical phenotype. Acta Neuropathol..

[97] Toledo J.B., Cairns N.J., Da X., Chen K., Carter D., Fleisher A., Householder E., Ayutyanont N., Roontiva A., Bauer R.J. (2013). Clinical and multimodal biomarker correlates of ADNI neuropathological findings. Acta Neuropathol. Commun..

[98] Jellinger K.A., Attems J. (2011). Prevalence and pathology of dementia with Lewy bodies in the oldest old: A comparison with other dementing disorders. Dement. Geriatr. Cogn. Disord..

[99] Hansen L.A., Masliah E., Galasko D., Terry R.D. (1993). Plaque-only Alzheimer disease is usually the lewy body variant, and vice versa. J. Neuropathol. Exp. Neurol..

[100] Schneider J.A., Arvanitakis Z., Leurgans S.E., Bennett D.A. (2009). The neuropathology of probable Alzheimer disease and mild cognitive impairment. Ann. Neurol..

[101] Olichney J.M., Galasko D., Salmon D.P., Hofstetter C.R., Hansen L.A., Katzman R., Thal L.J. (1998). Cognitive decline is faster in Lewybody variant than in Alzheimer’s disease. Neurology.

[102] Mathis C.A., Mason N.S., Lopresti B.J., Klunk W.E. (2012). Development of positron emission tomography beta-amyloid plaque imaging agents. Semin. Nucl. Med..

[103] Jagust W. (2014). Time for tau. Brain.

[104] Buchhave P., Minthon L., Zetterberg H., Wallin A.K., Blennow K., Hansson O. (2012). Cerebrospinal fluid levels of beta-amyloid 1–42, but not of tau, are fully changed already 5 to 10 years before the onset of Alzheimer dementia. Arch. Gen. Psychiatry.

[105] Fagan A.M., Xiong C., Jasielec M.S., Bateman R.J., Goate A.M., Benzinger T.L., Ghetti B., Martins R.N., Masters C.L., Mayeux R. (2014). Longitudinal change in CSF biomarkers in autosomal-dominant Alzheimer’s disease. Sci. Transl. Med..

[106] Ritchie C., Smailagic N., Noel-Storr A.H., Takwoingi Y., Flicker L., Mason S.E., McShane R. (2014). Plasma and cerebrospinal fluid amyloid beta for the diagnosis of Alzheimer’s disease dementia and other dementias in people with mild cognitive impairment (MCI). Cochrane Database Syst. Rev..

[107] Morris J.K., Honea R.A., Vidoni E.D., Swerdlow R.H., Burns J.M. (2014). Is Alzheimer’s disease a systemic disease?. Biochim. Biophys. Acta.

[108] Arvanitakis Z., Wilson R.S., Bienias J.L., Evans D.A., Bennett D.A. (2004). Diabetes mellitus and risk of Alzheimer disease and decline in cognitive function. Arch. Neurol..

[109] Luchsinger J.A., Reitz C., Patel B., Tang M.X., Manly J.J., Mayeux R. (2007). Relation of diabetes tomild cognitive impairment.. Arch. Neurol..

[110] Xu W., Qiu C., Gatz M., Pedersen N.L., Johansson B., Fratiglioni L. (2009). Mid- and late-life diabetes in relation to the risk of dementia: A population-based twin study. Diabetes.

[111] Hu W.T., Holtzman D.M., Fagan A.M., Shaw L.M., Perrin R., Arnold S.E., Grossman M., Xiong C., Craig-Schapiro R., Clark C.M. (2012). Plasma multianalyte profiling in mild cognitive impairment and Alzheimer disease. Neurology.

[112] Rosales-Corral S., Tan D.X., Manchester L., Reiter R.J. (2015). Diabetes and Alzheimer disease, two overlapping pathologies with the same background: Oxidative stress. Oxid. Med. Cell Longev..

[113] Williams T.L., Choi J.K., Surewicz K., Surewicz W.K. (2015). Soluble Prion Protein Binds Isolated Low Molecular Weight Amyloid-β Oligomers Causing Cytotoxicity Inhibition. ACS Chem. Neurosci..

[114] Giuffrida M.L., Caraci F., Pignataro B., Cataldo S., De Bona P., Bruno V., Molinaro G., Pappalardo G., Messina A., Palmigiano A. (2009). Beta-amyloid monomers are neuroprotective. J. Neurosci..

[115] Barger S.W., Harmon A.D. (1997). Microglial activation by Alzheimer amyloid precursor protein and modulation by apolipoprotein E. Nature.

[116] Meziane H., Dodart J.C., Mathis C., Little S., Clemens J., Paul S.M., Ungerer A. (1998). Memory-enhancing effects of secreted forms of the beta-amyloid precursor protein in normal and amnestic mice. Proc. Natl. Acad. Sci. USA.

[117] Tanzi R.E., Bertram L. (2005). Twenty years of the Alzheimer’s disease amyloid hypothesis: A genetic perspective. Cell.

[118] Parkin E.T., Hussain I., Karran E.H., Turner A.J., Hooper N.M. (1999). Characterization of detergent-insoluble complexes containing the familial Alzheimer’s disease-associated presenilins. J. Neurochem..

[119] Parkin E.T., Turner A.J., Hooper N.M. (1999). Amyloid precursor protein, although partially detergent-insoluble in mouse cerebral cortex, behaves as an atypical lipid raft protein. Biochem. J..

[120] Riddell D.R., Christie G., Hussain I., Dingwall C. (2001). Compartmentalization of beta-secretase (Asp2) into low-buoyant density, noncaveolar lipid rafts. Curr. Biol..

[121] Wahrle S., Das P., Nyborg A.C., McLendon C., Shoji M., Kawarabayashi T., Younkin L.H., Younkin S.G., Golde T.E. (2002). Cholesterol-dependent gamma-secretase activity in buoyant cholesterol-rich membrane microdomains. Neurobiol. Dis..

[122] Ehehalt R., Keller P., Haass C., Thiele C., Simons K. (2003). Amyloidogenic processing of the Alzheimer beta-amyloid precursor protein depends on lipid rafts. J. Cell Biol..

[123] Caporaso G.L., Gandy S.E., Buxbaum J.D., Ramabhadran T.V., Greengard P. (1992). Protein phosphorylation regulates secretion of Alzheimer beta/A4 amyloid precursor protein. Proc. Natl. Acad. Sci. USA.

[124] Savage M.J., Trusko S.P., Howland D.S., Pinsker L.R., Mistretta S., Reaume A.G., Greenberg B.D., Siman R., Scott R.W. (1998). Turnover of amyloid beta-protein in mouse brain and acute reduction of its level by phorbol ester. J. Neurosci..

[125] Van Nostrand W.E., Wagner S.L., Shankle W.D., Farrow J.S., Dick M., Rozemuller J.M., Kuiper M.A., Wolters E.C., Zimmerman J., Cotman C.W. (1992). Decreased levels of soluble amyloid beta-protein precursor in cerebrospinal fluid of live Alzheimer disease patients. Proc. Natl. Acad. Sci. USA.

[126] Lannfelt L., Basun H., Wahlund L.O., Rowe B.A., Wagner S.L. (1995). Decreased alpha-secretase-cleaved amyloid precursor protein as a diagnostic marker for Alzheimer’s disease. Decreased alpha-secretase-cleaved amyloid precursor protein as a diagnostic marker for Alzheimer’s disease. Nat. Med..

[127] Hooper N.M. (2005). Roles of proteolysis and lipid rafts in the processing of the amyloid precursor protein and prion protein. Biochem. Soc. Trans..

[128] Hardy J., Selkoe D.J. (2002). The amyloid hypothesis of Alzheimer’s disease: Progress and problems on the road to therapeutics. Science.

[129] Kojro E., Gimpl G., Lammich S., Marz W., Fahrenholz F. (2001). Low cholesterol stimulates the nonamyloidogenic pathway by its effect on the alpha -secretase ADAM 10. Proc. Natl. Acad. Sci. USA.

[130] Fassbender K., Simons M., Bergmann C., Stroick M., Lutjohann D., Keller P., Runz H., Kuhl S., Bertsch T., von Bergmann K. (2001). Simvastatin strongly reduces levels of Alzheimer’s disease beta-amyloid peptides Abeta 42 and Abeta 40 *in vitro* and *in vivo*. Proc. Natl. Acad. Sci. USA.

[131] Schellenberg G.D., Montine T.J. (2012). The genetics and neuropathology of Alzheimer’s disease. Acta Neuropathol..

[132] Takasugi N., Tomita T., Hayashi I., Tsuruoka M., Niimura M., Takahashi Y., Thinakaran G., Iwatsubo T. (2003). The role of presenilin cofactors in the γ-secratase complex. Nature.

[133] Kimberly W.T., LaVoie M.J., Ostaszewski B.L., Ye W., Wolfe M.S., Selkoe D.J. (2003). γ-Secretase is a membrane protein complex comprised of presenilin, nicastrin, aph-1, and pen-2. Proc. Natl. Acad. Sci. USA.

[134] Scheuner D., Eckman C., Jensen M., Song X., Citron M., Suzuki N., Bird T.D., Hardy J., Hutton M., Kukull W. (1996). Secreted amyloid beta-protein similar to that in the senile plaques of Alzheimer’s disease is increased *in vivo* by the presenilin 1 and 2 and APP mutations linked to familial Alzheimer’s disease. Nat. Med..

[135] Borchelt D.R., Thinakaran G., Eckman C.B., Lee M.K., Davenport F., Ratovitsky T., Prada C.M., Kim G., Seekins S., Yager D. (1996). Familial Alzheimer’s disease-linked presenilin I variants elevate Aβ1–42/1–40 ratio *in vitro* and *in vivo*. Neuron.

[136] Funk K.E., Kuret J. (2012). Lysosomal fusion dysfunction as a unifying hypothesis for Alzheimer’s disease pathology. Int. J. Alzheimers Dis..

[137] Gu Z., Liu W., Yan Z. (2009). β-Amyloid impairs AMPA receptor trafficking and function by reducing Ca^2+^/calmodulin-dependent protein kinase II synaptic distribution. J. Biol. Chem..

[138] Tyszkiewicz J.P., Yan Z. (2005). β-Amyloid peptides impair PKC-dependent functions of metabotropic glutamate receptors in prefrontal cortical neurons. J. Neurophysiol..

[139] Laurén J., Gimbel D.A., Nygaard H.B., Gilbert J.W., Stephen M. (2009). Cellular prion protein mediates impairment of synaptic plasticity by amyloid-B oligomers. Nature.

[140] Renner M., Lacor P.N., Velasco P.T., Xu J., Contractor A., Klein W.L., Triller A. (2010). Deleterious effects of amyloid beta oligomers acting as an extracellular scaffold for mGluR5. Neuron.

[141] Gimbel D.A., Nygaard H.B., Coffey E.E., Gunther E.C., Laurén J., Gimbel Z.A., Strittmatter S.M. (2010). Memory impairment in transgenic Alzheimer mice requires cellular prion protein. J. Neurosci..

[142] Barry A.E., Klyubin I., Mc Donald J.M., Mably A.J., Farrell M.A., Scott M., Walsh D.M., Rowan M.J. (2011). Alzheimer’s disease brain-derived Amyloid-β-mediated inhibition of LTP *in vivo* is prevented by immunotargeting cellular prion protein. J. Neurosci..

[143] Balducci C., Beeg M., Stravalaci M., Bastone A., Sclip A., Biasini E., Tapella L., Colombo L., Manzoni C., Borsello T. (2010). Synthetic amyloid-beta oligomers impair long-term memory independently of cellular prion protein. Proc. Natl. Acad. Sci. USA.

[144] Calella A.M., Farinelli M., Nuvolone M., Mirante O., Moos R., Falsig J., Mansuy I.M., Aguzzi A. (2010). Prion protein and Abeta-related synaptic toxicity impairment. EMBO Mol. Med..

[145] Cissé M., Sanchez P.E., Kim D.H., Ho K., Yu G.Q., Mucke L. (2011). Ablation of cellular prion protein does not ameliorate abnormal neural network activity or cognitive dysfunction in the J20 line of human amyloid precursor protein transgenic mice. J. Neurosci..

[146] Resenberger U.K., Harmeier A., Woerner A.C., Goodman J.L., Müller V., Krishnan R., Vabulas R.M., Kretzschmar H.A., Lindquist S., Hartl F.U. (2011). The cellular prion protein mediates neurotoxic signalling of β-sheet-rich conformers independent of prion replication. EMBO J..

[147] Freir D.B., Nicoll A.J., Klyubin I., Panico S., Mc Donald J.M., Risse E., Asante E.A., Farrow M.A., Sessions R.B., Saibil H.R. (2011). Interaction between prion protein and toxic amyloid β assemblies can be therapeutically targeted at multiple sites. Nat. Commun..

[148] Hamilton A., Zamponi G.W., Ferguson S.S. (2015). Glutamate receptors function as scaffolds for the regulation of β-amyloid and cellular prion protein signaling complexes. Mol. Brain.

[149] Stys P.K., You H., Zamponi G.W. (2012). Copper-dependent regulation of NMDA receptors by cellular prion protein: Implications for neurodegenerative disorders. J. Physiol..

[150] Khosravani H., Zhang Y., Tsutsui S., Hameed S., Altier C., Hamid J., Chen L., Villemaire M., Ali Z., Jirik F.R. (2008). Prion protein attenuates excitotoxicity by inhibiting NMDA receptors. J. Cell Biol..

[151] Gandy S. (2005). The role of cerebral amyloid beta accumulation in common forms of Alzheimer disease. J. Clin. Investig..

[152] Necula M., Kayed R., Milton S., Glabe C.G. (2007). Small molecule inhibitors of aggregation indicate that amyloid beta oligomerization and fibrillization pathways are independent and distinct. J. Biol. Chem..

[153] Butterfield D.A., Galvan V., Lange M.B., Tang H., Sowell R.A., Spilman P., Fombonne J., Gorostiza O., Zhang J., Sultana R. (2010). *In vivo* oxidative stress in brain of Alzheimer disease transgenic mice: Requirement for methionine 35 in amyloid beta-peptide of APP. Free Radic. Biol. Med..

[154] Al-Hilaly Y.K., Williams T.L., Stewart-Parker M., Ford L., Skaria E., Cole M., Bucher W.G., Morris K.L., Sada A.A., Thorpe J.R. (2013). A central role for dityrosine crosslinking of Amyloid-β in Alzheimer’s disease. Acta Neuropathol. Commun..

[155] Drewes G., Ebneth A., Mandelkow E.M. (1998). MAPs, MARKs and microtubule dynamics. Trends Biochem. Sci..

[156] Kosik K.S., McConlogue L. (1994). Microtubule-associated protein function: Lessons from expression in Spodoptera frugiperda cells. Cell Motil. Cytoskeleton..

[157] Illenberger S., Zheng-Fischhöfer Q., Preuss U., Stamer K., Baumann K., Trinczek B., Biernat J., Godemann R., Mandelkow E.M., Mandelkow E. (1998). The endogenous and cell cycle-dependent phosphorylation of tau protein in living cells: Implications for Alzheimer’s disease. Mol. Biol. Cell.

[158] Mandelkow E.M., Biernat J., Drewes G., Gustke N., Trinczek B., Mandelkow E. (1995). Tau domains, phosphorylation, and interactions with microtubules. Neurobiol. Aging.

[159] Trojanowski J.Q., Lee V.M. (1995). Phosphorylation of paired helical filament tau in Alzheimer’s disease neurofibrillary lesions: Focusing on phosphatases. FASEB J..

[160] Delacourte A., Buée L. (1997). Normal and pathological Tau proteins as factors for microtubule assembly. Int. Rev. Cytol..

[161] Mandelkow E.M., Mandelkow E. (1998). Tau in Alzheimer’s disease. Trends Cell Biol..

[162] Hyman B.T., Phelps C.H., Beach T.G., Bigio E.H., Cairns N.J., Carrillo M.C., Dickson D.W., Duyckaerts C., Frosch M.P., Masliah E. (2012). National Institute on Aging-Alzheimer’s Association guidelines for the neuropathologic assessment of Alzheimer’s disease. Alzheimers Dement..

[163] Goedert M., Jakes R., Spillantini M.G., Hasegawa M., Smith M.J., Crowther R.A. (1996). Assembly of microtubule-associated protein tau into Alzheimer-like filaments induced by sulphated glycosaminoglycans. Nature.

[164] Kampers T., Friedhoff P., Biernat J., Mandelkow E.M., Mandelkow E. (1996). RNA stimulates aggregation of microtubule-associated protein tau into Alzheimer-like paired helical filaments. FEBS Lett..

[165] Novak M., Kabat J., Wischik C.M. (1993). Molecular characterization of the minimal protease resistant tau unit of the Alzheimer’s disease paired helical filament. EMBO J..

[166] Yan S.D., Chen X., Schmidt A.M., Brett J., Godman G., Zou Y.S., Scott C.W., Caputo C., Frappier T., Smith M.A. (1994). Glycated tau protein in Alzheimer disease: A mechanism for induction of oxidant stress. Proc. Natl. Acad. Sci. USA.

[167] Arriagada P.V., Growdon J.H., Hedley-Whyte E.T., Hyman B.T. (1992). Neurofibrillary tangles but not senile plaques parallel duration and severity of Alzheimer’s disease. Neurology.

[168] Nelson P.T., Alafuzoff I., Bigio E.H., Bouras C., Braak H., Cairns N.J., Castellani R.J., Crain B.J., Davies P., Del Tredici K. (2012). Correlation of Alzheimer disease neuropathologic changes with cognitive status: A review of the literature. J. Neuropathol. Exp. Neurol..

[169] Hyman B.T., Marzloff K., Arriagada P.V. (1993). The lack of accumulation of senile plaques or amyloid burden in Alzheimer’s disease suggests a dynamic balance between amyloid deposition and resolution. J. Neuropathol. Exp. Neurol..

[170] Ishiki A., Okamura N., Furukawa K., Furumoto S., Harada R., Tomita N., Hiraoka K., Watanuki S., Ishikawa Y., Tago T. (2015). Longitudinal Assessment of Tau Pathology in Patients with Alzheimer’s Disease Using [18F]THK-5117 Positron Emission Tomography. PLoS ONE.

[171] Plácido A.I., Pereira C.M., Duarte A.I., Candeias E., Correia S.C., Santos R.X., Carvalho C., Cardoso S., Oliveira C.R., Moreira P.I. (2014). The role of endoplasmic reticulum in amyloid precursor protein processing and trafficking: Implications for Alzheimer’s disease. Biochim. Biophys. Acta.

[172] Chang R.C., Wong A.K., Ng H.K., Hugon J. (2002). Phosphorylation of eukaryotic initiation factor-2alpha (eIF2alpha) is associated with neuronal degeneration in Alzheimer’s disease. Neuroreport.

[173] O’Connor T., Sadleir K.R., Maus E., Velliquette R.A., Zhao J., Cole S.L., Eimer W.A., Hitt B., Bembinster L.A., Lammich L. (2008). Phosphorylation of the translation initiation factor eIF2alpha increases BACE1 levels and promotes amyloidogenesis. Neuron.

[174] Honjo Y., Ito H., Horibe T., Takahashi R., Kawakami K. (2010). Protein disulfide isomerase-immunopositive inclusions in patients with Alzheimer disease. Brain Res..

[175] Lee J.H., Won S.M., Suh J., Son S.J., Moon G.J., Park U.J., Gwag B.J. (2010). Induction of the unfolded protein response and cell death pathway in Alzheimer’s disease, but not in aged Tg2576 mice. Exp. Mol. Med..

[176] Kaneko M., Koike H., Saito R., Kitamura Y., Okuma Y., Nomura Y. (2010). Loss of HRD1-mediated protein degradation causes amyloid precursor protein accumulation and amyloid-beta generation. J. Neurosci..

[177] Hoozemans J.J., Veerhuis R., van Haastert E.S., Rozemuller J.M., Baas F., Eikelenboom P., Scheper W. (2005). The unfolded protein response is activated in Alzheimer’s disease. Acta Neuropathol..

[178] Katayama T., Imaizumi K., Sato N., Miyoshi K., Kudo T., Hitomi J., Morihara T., Yoneda T., Gomi F., Mori Y. (1999). Presenilin-1 mutations downregulate the signalling pathway of the unfolded-protein response. Nat. Cell Biol..

[179] Hamos J.E., Oblas B., Pulaski-Salo D., Welch W.J., Bole D.G., Drachman D.A. (1991). Expression of heat shock proteins in Alzheimer’s disease. Neurology.

[180] Unterberger U., Hoftberger R., Gelpi E., Flicker H., Budka H., Voigtlander T. (2006). Endoplasmic reticulum stress features are prominent in Alzheimer disease but not in prion diseases *in vivo*. J. Neuropathol. Exp. Neurol..

[181] Costa R.O., Ferreiro E., Cardoso S.M., Oliveira C.R., Pereira C.M. (2010). ER stressmediated apoptotic pathway induced by Abeta peptide requires the presence of functional mitochondria. J. Alzheimers Dis..

[182] Resende R., Ferreiro E., Pereira C., Oliveira C.R. (2008). ER stress is involved in Abetainduced GSK-3beta activation and tau phosphorylation. J. Neurosci. Res..

[183] Song S., Lee H., Kam T.I., Tai M.L., Lee J.Y., Noh J.Y., Shim S.M., Seo S.J., Kong Y.Y., Nakagawa T. (2008). E2–25K/Hip-2 regulates caspase-12 in ER stress-mediated Abeta neurotoxicity. J. Cell Biol..

[184] Milhavet O., Martindale J.L., Camandola S., Chan S.L., Gary D.S., Cheng A., Holbrook N.J., Mattson M.P. (2002). Involvement of Gadd153 in the pathogenic action of presenilin-1 mutations. J. Neurochem..

[185] Marwarha G., Raza S., Prasanthi J.R., Ghribi O. (2013). Gadd153 and NF-kappaB crosstalk regulates 27-hydroxycholesterol-induced increase in BACE1 and beta-amyloid production in human neuroblastoma SH-SY5Y cells. PLoS ONE.

[186] Hoozemans J.J., van Haastert E.S., Nijholt D.A., Rozemuller A.J., Eikelenboom P., Scheper W. (2009). The unfolded protein response is activated in pretangle neurons in Alzheimer’s disease hippocampus. Am. J. Pathol..

[187] Hou H.L., Shen Y.X., Zhu H.Y., Sun H., Yan X.B., Fang H., Zhou J.N. (2006). Alterations of hHrd1 expression are related to hyperphosphorylated tau in the hippocampus in Alzheimer’s disease. J. Neurosci. Res..

[188] Van der Harg J.M., Nölle A., Zwart R., Boerema A.S., van Haastert E.S., Strijkstra A.M., Hoozemans J.J., Scheper W. (2014). The unfolded protein response mediates reversible tau phosphorylation induced by metabolic stress. Cell Death Dis..

[189] Abisambra J.F., Jinwal U.K., Blair L.J., O’Leary J.C., Li Q., Brady S., Wang L., Guidi C.E., Zhang B., Nordhues B.A. (2013). Tau accumulation activates the unfolded protein response by impairing endoplasmic reticulum-associated degradation. J. Neurosci..

[190] Nixon R.A. (2007). Autophagy, amyloidogenesis and Alzheimer disease. J. Cell Sci..

[191] Pickford F., Masliah E., Britschgi M., Lucin K., Narasimhan R., Jaeger P.A., Small S., Spencer B., Rockenstein E., Levine B. (2008). The autophagy-related protein beclin 1 shows reduced expression in early Alzheimer disease and regulates amyloid beta accumulation in mice. J. Clin. Investig..

[192] Salminen A., Kaarniranta K., Kauppinen A., Ojala J., Haapasalo A., Soininen H., Hiltunen M. (2013). Impaired autophagy and APP processing in Alzheimer’s disease: The potential role of Beclin 1 interactome. Prog. Neurobiol..

[193] Friedman L.G., Qureshi Y.H., Yu W.H. (2014). Promoting Autophagic Clearance: Viable Therapeutic Targets in Alzheimer’s Disease. Neurotherapeutics.

[194] Zhou F., Chen S., Xiong J., Li Y., Qu L. (2012). Luteolin reduces zinc-induced tau phosphorylation at Ser262/356 in an ROS-dependent manner in SH-SY5Y cells. Biol. Trace Elem. Res..

[195] Eckert A., Nisbet R., Grimm A., Götz J. (2014). March separate, strike together--role of phosphorylated TAU in mitochondrial dysfunction in Alzheimer’s disease. Biochim. Biophys. Acta.

[196] Rhein V., Song X., Wiesner A., Ittner L.M., Baysang G., Meier F., Ozmen L., Bluethmann H., Dröse S., Brandt U. (2009). Amyloid-beta and tau synergistically impair the oxidative phosphorylation system in triple transgenic Alzheimer’s disease mice. Proc. Natl. Acad. Sci. USA.

[197] Casley C.S., Canevari L., Land J.M., Clark J.B., Sharpe M.A. (2002). β-Amyloid inhibits integrated mitochondrial respiration and key enzyme activities. J. Neurochem..

[198] Maurer I., Zierz S., Moller H.J. (2000). A selective defect of cytochrome c oxidase is present in brain of Alzheimer disease patients. Neurobiol. Aging.

[199] Hely M.A., Reid W.G., Adena M.A., Halliday G.M., Morris J.G. (2008). The Sydney multicenter study of Parkinson’s disease: The inevitability of dementia at 20 years. Mov. Disord..

[200] Kokhan V.S., Afanasyeva M.A., Van’kin G.I. (2012). α-Synuclein knockout mice have cognitive impairments. Behav. Brain Res..

[201] Schneider J.A., Arvanitakis Z., Yu L., Boyle P.A., Leurgans S.E., Bennett D.A. (2012). Cognitive impairment, decline and fluctuations in older community-dwelling subjectswith Lewy bodies. Brain.

[202] Spillantini M.G., Schmidt M.L., Lee V.M.-Y., Trojanowski J.Q., Jakes R., Goedert M. (1997). α-Synuclein in Lewy bodies. Nature.

[203] Spillantini M.G., Crowther R.A., Jakes R., Hasegawa M., Goedert M. (1998). α-Synuclein in filamentous inclusions of Lewy bodies from Parkinson’s disease and dementia with Lewy bodies. Proc. Natl. Acad. Sci. USA.

[204] Engelender S. (2008). Ubiquitination of alpha-synuclein and autophagy in Parkinson’s disease. Autophagy.

[205] Ishizawa T., Mattila P., Davies P., Wang D., Dickson D.W. (2003). Colocalization of tau and alpha-synuclein epitopes in Lewy bodies. J. Neuropathol. Exp. Neurol..

[206] Arima K., Hirai S., Sunohara N., Aoto K., Izumiyama Y., Uéda K., Ikeda K., Kawai M., Arima K., Hirai S. (1999). Cellular co-localization of phosphorylated tau- and NACP/alpha-synuclein-epitopes in Lewy bodies in sporadic Parkinson’s disease and in dementia with Lewy bodies. Brain Res..

[207] Singleton A.B., Farrer M., Johnson J., Singleton A., Hague S., Kachergus J., Hulihan M., Peuralinna T., Dutra A., Nussbaum R. (2003). α-Synuclein locus triplication causes Parkinson’s disease. Science.

[208] Polymeropoulos M.H., Lavedan C., Leroy E., Ide S.E., Dehejia A., Dutra A., Pike B., Root H., Rubenstein J., Boyer R. (1997). Mutation in the α-synuclein gene identified in families with Parkinson’s disease. Science.

[209] Krüger R., Kuhn W., Müller T., Woitalla D., Graeber M., Kösel S., Przuntek H., Epplen J.T., Schöls L., Riess O. (1998). Ala30Pro mutation in the gene encoding α-synuclein in Parkinson’s disease. Nat. Genet..

[210] Zarranz J.J., Alegre J., Gómez-Esteban J.C., Lezcano E., Ros R., Ampuero I., Vidal L., Hoenicka J., Rodriguez O., Atarés B. (2004). The new mutation, E46K, of α-synuclein causes Parkinson and Lewy body dementia. Ann. Neurol..

[211] Clayton D.F., George J.M. (1998). The synucleins: A family of proteins involved in synaptic function, plasticity, neurodegeneration and disease. Trends Neurosci..

[212] Clayton D.F., George J.M. (1999). Synucleins in synaptic plasticity and neurodegenerative disorders. J. Neurosci. Res..

[213] Spencer B., Potkar R., Trejo M., Rockenstein E., Patrick C., Gindi R., Adame A., Wyss-Coray T., Masliah E. (2009). Beclin 1 gene transfer activates autophagy and ameliorates the neurodegenerative pathology in a-synuclein models of Parkinson’s and Lewy body diseases. J. Neurosci..

[214] Fortin D.L., Nemani V.M., Voglmaier S.M., Anthony M.D., Ryan T.A., Edwards R.H. (2005). Neural activity controls the synaptic accumulation of a-synuclein. J. Neurosci..

[215] Yu S., Li X., Liu G., Han J., Zhang C., Li Y., Xu S., Liu C., Gao Y., Yang H. (2007). Extensive nuclear localization of alpha-synuclein in normal rat brain neurons revealed by a novel monoclonal antibody. Neuroscience.

[216] Liu G., Zhang C., Yin J., Li X., Cheng F., Li Y., Yang H., Uéda K., Chan P., Yu S. (2009). alpha-Synuclein is differentially expressed in mitochondria from different rat brain regions and dose-dependently down-regulates complex I activity. Neurosci. Lett..

[217] Alim M.A., Hossain M.S., Arima K., Takeda K., Izumiyama Y., Nakamura M., Kaji H., Shinoda T., Hisanaga S., Ueda K. (2002). Tubulin seeds alpha-synuclein fibril formation. J. Biol. Chem..

[218] Alim M.A., Ma Q.L., Takeda K., Aizawa T., Matsubara M., Nakamura M., Asada A., Saito T., Kaji H., Yoshii M. (2004). Demonstration of a role for alpha-synuclein as a functional microtubule-associated protein. J. Alzheimers Dis..

[219] Cooper A.A., Gitler A.D., Cashikar A., Haynes C.M., Hill K.J., Bhullar B., Liu K., Xu K., Strathearn K.E., Liu F. (2006). Alpha-synuclein blocks ER-Golgi traffic and Rab1 rescues neuron loss in Parkinson’s models. Science.

[220] Martinez-Vicente M., Talloczy Z., Kaushik S., Massey A.C., Mazzulli J., Mosharov E.V., Hodara R., Fredenburg R., Wu D.C., Follenzi A. (2008). Dopamine-modified a-synuclein blocks chaperone-mediated autophagy. J. Clin. Investig..

[221] Esteves A.R., Arduíno D.M., Swerdlow R.H., Oliveira C.R., Cardoso S.M. (2010). Microtubule depolymerization potentiates α-synuclein oligomerization. Front. Aging Neurosci..

[222] Kim M., Jung W., Lee I.H., Bhak G., Paik S.R., Hahn J.S. (2008). Impairment of microtubule system increases α-synuclein aggregation and toxicity. Biochem. Biophys. Res. Commun..

[223] Lee H.J., Khoshaghideh F., Lee S., Lee S.J. (2006). Impairment of microtubule-dependent trafficking by overexpression of α-synuclein. Eur. J. Neurosci..

[224] Nakayama K., Suzuki Y., Yazawa I. (2009). Microtubule depolymerization suppresses α-synuclein accumulation in a mouse model of multiple system atrophy. Am. J. Pathol..

[225] Nakayama K., Suzuki Y., Yazawa I. (2012). Binding of neuronal α-synuclein to β-III tubulin and accumulation in a model of multiple system atrophy. Biochem. Biophys. Res. Commun..

[226] Zhou R.M., Huang Y.X., Li X.L., Chen C., Shi Q., Wang G.R., Tian C., Wang Z.Y., Jing Y.Y., Gao C., Dong X.P. (2010). Molecular interaction of α-synuclein with tubulin influences on the polymerization of microtubule *in vitro* and structure of microtubule in cells. Mol. Biol. Rep..

[227] Osterberg V.R., Spinelli K.J., Weston L.J., Luk K.C., Woltjer R.L., Unni V.K. (2015). Progressive aggregation of alpha-synuclein and selective degeneration of lewy inclusion-bearing neurons in a mouse model of parkinsonism. Cell Rep..

[228] Jucker M., Walker L.C. (2013). Self-propagation of pathogenic protein aggregates in neurodegenerative diseases. Nature.

[229] Guo J.L., Lee V.M. (2014). Cell-to-cell transmission of pathogenic proteins in neurodegenerative diseases. Nat. Med..

[230] Fraser P.E. (2014). Prions and prion-like proteins. J. Biol. Chem..

[231] Tofaris G.K., Spillantini M.G. (2007). Physiological and pathological properties of alpha-synuclein. Cell Mol. Life Sci..

[232] Ross O.A., Braithwaite A.T., Skipper L.M., Kachergus J., Hulihan M.M., Middleton F.A., Nishioka K., Fuchs J., Gasser T., Maraganore D.M. (2008). Genomic investigation of alpha-synuclein multiplication and parkinsonism. Ann. Neurol..

[233] Chung C.Y., Khurana V., Auluck P.K., Tardiff D.F., Mazzulli J.R., Soldner F., Baru V., Lou Y., Freyzon Y., Cho S. (2013). Identification and rescue of α-synuclein toxicity in Parkinson patient-derived neurons. Science.

[234] Gorbatyuk M.S., Shabashvili A., Chen W., Meyers C., Sullivan L.F., Salganik M., Lin J.H., Lewin A.S., Muzyczka N., Gorbatyuk O.S. (2012). Glucose regulated protein 78 diminishes α-synuclein neurotoxicity in a rat model of Parkinson disease. Mol. Ther..

[235] Hashida K., Kitao Y., Sudo H., Awa Y., Maeda S., Mori K., Takahashi R., Iinuma M., Hori O. (2012). ATF6alpha promotes astroglial activation and neuronal survival in a chronic mouse model of Parkinson’s disease. PLoS ONE.

[236] Egawa N., Yamamoto K., Inoue H., Hikawa R., Nishi K., Mori K., Takahashi R. (2011). The endoplasmic reticulum stress sensor, ATF6α, protects against neurotoxin-induced dopaminergic neuronal death. J. Biol. Chem..

[237] Boyce M., Bryant K.F., Jousse C., Long K., Harding H.P., Scheuner D., Kaufman R.J., Ma D., Coen D.M., Ron D. (2005). A selective inhibitor of eIF2alpha dephosphorylation protects cells from ER stress. Science.

[238] Colla E., Coune P., Liu Y., Pletnikova O., Troncoso J.C., Iwatsubo T., Schneider B.L., Lee M.K. (2012). Endoplasmic reticulum stress is important for the manifestations of α-synucleinopathy *in vivo*. J. Neurosci..

[239] Credle J.J., Forcelli P.A., Delannoy M., Oaks A.W., Permaul E., Berry D.L., Duka V., Wills J., Sidhu A. (2015). α-Synuclein-mediated inhibition of ATF6 processing into COPII vesicles disrupts UPR signaling in Parkinson’s disease. Neurobiol. Dis..

[240] Hoozemans J.J., van Haastert E.S., Eikelenboom P., de Vos R.A., Rozemuller J.M., Scheper W. (2007). Activation of the unfolded protein response in Parkinson’s disease. Biochem. Biophys. Res. Commun..

[241] Makioka K., Yamazaki T., Fujita Y., Takatama M., Nakazato Y., Okamoto K. (2010). Involvement of endoplasmic reticulum stress defined by activated unfolded protein response in multiple system atrophy. J. Neurol. Sci..

[242] Stefanis L., Larsen K.E., Rideout H.J., Sulzer D., Greene L.A. (2001). Expression of A53T mutant but not wild-type alpha-synuclein in PC12 cells induces alterations of the ubiquitin-dependent degradation system, loss of dopamine release, and autophagic cell death. J. Neurosci..

[243] Stefanis L. (2012). α-Synuclein in Parkinson’s disease. Cold Spring Harb. Perspect. Med..

[244] Hokenson M.J., Uversky V.N., Goers J., Yamin G., Munishkina L.A., Fink A.L. (2004). Role of individual methionines in the fibrillation of methionine-oxidized alphasynuclein. Biochemistry.

[245] Leong S.L., Pham C.L., Galatis D., Fodero-Tavoletti M.T., Perez K., Hill A.F., Masters C.L., Ali F.E., Barnham K.J., Cappai R. (2009). Formation of dopamine-mediated alpha-synuclein-soluble oligomers requires methionine oxidation. Free Radic. Biol. Med..

[246] Winslow A.R., Chen C.-W., Corrochano S., Acevedo-Arozena A., Gordon D.E., Peden A.A., Lichtenberg M., Menzies F.M., Ravikumar B., Imarisio S. (2010). α-Synuclein impairs macroautophagy: Implications for Parkinson’s disease. J. Cell Biol..

[247] Schapira A.H. (2008). Mitochondria in the aetiology and pathogenesis of Parkinson’s disease. Lancet Neurol..

[248] Sherer T.B., Betarbet R., Stout A.K., Lund S., Baptista M., Panov A.V., Cookson M.R., Greenamyre J.T. (2002). An *in vitro* model of Parkinson’s disease: Linking mitochondrial impairment to altered alpha-synuclein metabolism and oxidative damage. J. Neurosci..

[249] Burchell V.S., Nelson D.E., Sanchez-Martinez A., Delgado-Camprubi M., Ivatt R.M., Pogson J.H., Randle S.J., Wray S., Lewis P.A., Houlden H. (2013). The Parkinson’s disease-linked proteins Fbxo7 and Parkin interact to mediate mitophagy. Nat. Neurosci..

[250] Chen Y., Dorn G.W. (2013). PINK1-phosphorylated mitofusin 2 is a Parkin receptor for culling damaged mitochondria. Science.

[251] Yang J.Y., Yang W.Y. (2013). Bit-by-bit autophagic removal of parkin-labelled mitochondria. Nat. Commun..

[252] Kuroda Y., Mitsui T., Kunishige M., Shono M., Akaike M., Azuma H., Matsumoto T. (2006). Parkin enhances mitochondrial biogenesis in proliferating cells. Hum. Mol. Genet..

[253] Rothfuss O., Fischer H., Hasegawa T., Maisel M., Leitner P., Miesel F., Sharma M., Bornemann A., Berg D., Gasser T., Patenge N. (2009). Parkin protects mitochondrial genome integrity and supports mitochondrial DNA repair. Hum. Mol. Genet..

[254] Shin J.H., Ko H.S., Kang H., Lee Y., Lee Y.I., Pletinkova O., Troconso J.C., Dawson V.L., Dawson T.M. (2011). PARIS (ZNF746) repression of PGC-1alpha contributes to neurodegeneration in Parkinson’s disease. Cell.

[255] Ding W.X., Yin X.M. (2012). Mitophagy: Mechanisms, pathophysiological roles, and analysis. Biol. Chem..

[256] Moreira P.I., Carvalho C., Zhu X., Smith M.A., Perry G. (2010). Mitochondrial dysfunction is a trigger of Alzheimer’s disease pathophysiology. Biochim. Biophys. Acta..

[257] Suen D.F., Narendra D.P., Tanaka A., Manfredi G., Youle R.J. (2010). Parkin overexpression selects against a deleterious mtDNA mutation in heteroplasmic cybrid cells. Proc. Natl. Acad. Sci. USA.

[258] Jin S.M., Lazarou M., Wang C., Kane L.A., Narendra D.P., Youle R.J. (2010). Mitochondrial membrane potential regulates PINK1 import and proteolytic destabilization by PARL. J. Cell Biol..

[259] Lazarou M., Jin S.M., Kane L.A., Youle R.J. (2012). Role of PINK1 binding to the TOM complex and alternate intracellular membranes in recruitment and activation of the E3 ligase Parkin. Dev. Cell.

[260] Fimia G.M., Stoykova A., Romagnoli A., Giunta L., Di Bartolomeo S., Nardacci R., Corazzari M., Fuoco C., Ucar A., Schwartz P. (2007). Ambra1 regulates autophagy and development of the nervous system. Nature.

[261] van Humbeeck C., Cornelissen T., Hofkens H., Mandemakers W., Gevaert K., de Strooper B., Vandenberghe W. (2011). Parkin interacts with Ambra1 to induce mitophagy. J. Neurosci..

[262] Hickson-Bick D.L., Jones C., Buja L.M. (2008). Stimulation of mitochondrial biogenesis and autophagy by lipopolysaccharide in the neonatal rat cardiomyocyte protects against programmed cell death. J. Mol. Cell Cardiol..

[263] Dagda R.K., Zhu J., Kulich S.M., Chu C.T. (2008). Mitochondrially localized ERK2 regulates mitophagy and autophagic cell stress: Implications for Parkinson’s disease. Autophagy.

[264] Kubli D.A., Gustafsson A.B. (2012). Mitochondria and mitophagy: The yin and yang of cell death control. Circ. Res..

[265] Yan J., Feng Z., Liu J., Shen W., Wang Y., Wertz K., Weber P., Long J. (2012). Enhanced autophagy plays a cardinal role in mitochondrial dysfunction in type 2 diabetic Goto-Kakizaki (GK) rats: Ameliorating effects of (−)-epigallocatechin-3-gallate. J. Nutr. Biochem..

[266] Zhu J.H., Horbinski C., Guo F., Watkins S., Uchiyama Y., Chu C.T. (2007). Regulation of autophagy by extracellular signal-regulated protein kinases during 1-methyl-4- phenylpyridinium-induced cell death. Am. J. Pathol..

[267] Kovacs G.G., Budka H. (2008). Prion diseases: From protein to cell pathology. Am. J. Pathol..

[268] Cuille J., Chelle P.L. (1936). Pathologie animale, la maladie dite tremblant du mouton est-elle inoculable. Compt. Rend. Acad. Sci..

[269] Dickinson A.G. (1976). Scrapie in sheep and goats. Front. Biol..

[270] Wells G.A., Scott A., Johnson C., Gunning R., Hancock R., Jeffrey M., Dawson M., Bradley R. (1987). A novel progressive spongiform encephalopathy in cattle. Vet. Rec..

[271] Williams E.S., Young S. (1982). Spongiform encephalopathy of Rocky Mountain elk. J. Wildl. Dis..

[272] Liberski P.P., Guiroy D.C., Williams E.S., Walis A., Budka H. (2001). Deposition patterns of disease-associated prion protein in captive mule deer brains with chronic wasting disease. Acta Neuropathol..

[273] Wyatt J.M., Pearson G.R., Smerdon T.N., Gruffydd-Jones T.J., Wells G., Wilesmith J.W. (1991). Naturally occurring scrapie-like spongiform encephalopathy in five domestic cats. Vet. Rec..

[274] Gajdusek D.C., Gibbs C.J., Alpers M. (1966). Experimental transmission of a Kuru-like syndrome to chimpanzees. Nature.

[275] Liberski P.P., Gajdusek D.C. (1997). Kuru: Forth years later, a historical note. Brain Pathol..

[276] Gibbs C., Gajdusek D.C., Asher D.M., Alpers M., Beck E., Daniel P.M., Matthews W.B. (1968). Creutzfeldt-Jakob disease (spongiform encephalopathy): Transmission to the chimpanzee. Science.

[277] Masters C.L., Gajdusek D.C., Gibbs C.J. (1981). Creutzfeldt-Jakob disease virus isolations from the Gerstmann–Stra¨ussler syndrome with an analysis of the various forms of amyloid plaque deposition in the virus-induced spongiform encephalopathies. Brain.

[278] Lugaresi E., Medori R., Montagna P., Baruzzi A., Cortelli P., Lugaresi A., Tinuper P., Zucconi M., Gambetti P. (1986). Fatal familial insomnia and dysautonomia with selective degeneration of thalamic nuclei. N. Engl. J. Med..

[279] Llewelyn C.A., Hewitt P.E., Knight R.S., Amar K., Cousens S., Mackenzie J., Will R.G. (2004). Possible transmission of variant Creutzfeldt-Jakob disease by blood transfusion. Lancet.

[280] Ironside J.W. (2006). Variant Creutzfeldt-Jakob disease: Risk of transmission by blood transfusion and blood therapies. Haemophilia.

[281] Godsave S.F., Peters P.J., Wille H. (2015). Subcellular distribution of the prion protein in sickness and in health. Virus Res..

[282] Prusiner S.B. (1998). Prions. Proc. Natl. Acad. Sci. USA.

[283] Brandner S., Isenmann S., Raeber A., Fischer M., Sailer A., Kobayashi Y., Marino S., Weissmann C., Aguzzi A. (1996). Normal host prion protein necessary forscrapie-induced neurotoxicity. Nature.

[284] Mallucci G., Dickinson A., Linehan J., Klohn P.C., Brandner S., Collinge J. (2003). Depleting neuronal PrP in prion infection prevents disease and reverses spon-giosis. Science.

[285] Larson M., Sherman M.A., Amar F., Nuvolone M., Schneider J.A., Bennett D.A., Aguzzi A., Lesne S.E. (2012). The complex PrP(c)-Fyn couples human oligomericAbeta with pathological tau changes in Alzheimer’s disease. J. Neurosci..

[286] Aguzzi A. (2008). Unraveling prion strains with cell biology and organic chemistry. Proc. Natl. Acad. Sci. USA.

[287] Borchelt D.R., Scott M., Taraboulos A., Stahl N., Prusiner S.B. (1990). Scrapie and cellular prion proteins differ in their kinetics of synthesis and topology in cultured-cells. J. Cell Biol..

[288] Caughey B., Raymond G.J. (1991). The scrapie-associated form of PrP is made from a cellsurface precursor that is both protease-sensitive and phospholipase-sensitive. J. Biol. Chem..

[289] Goold R., Rabbanian S., Sutton L., Andre R., Arora P., Moonga J., Clarke A.R., Schiavo G., Jat P., Collinge J. (2011). Rapid cell-surface prion protein conversion revealed using a novel cell system. Nat. Commun..

[290] Beranger F., Mange A., Goud B., Lehmann S. (2002). Stimulation of PrPC retrograde transport toward the endoplasmic reticulum increases accumulation of PrPSc in prioninfected cells. J. Biol. Chem..

[291] Borchelt D.R., Taraboulos A., Prusiner S.B. (1992). Evidence for synthesis of scrapie prion proteins in the endocytic pathway. J. Biol. Chem..

[292] Caughey B., Raymond G.J., Ernst D., Race R.E. (1991). *N*-terminal truncation of the scrapie-associated form of PrP by lysosomal protease(s)—Implications regarding the site of conversion of PrP to the protease-resistant state. J. Virol..

[293] Marijanovic Z., Caputo A., Campana V., Zurzolo C. (2009). Identification of an intracellular site of prion conversion. PLoS Pathog..

[294] Yamasaki T., Baron G.S., Suzuki A., Hasebe R., Horiuchi M. (2014). Characterization of intracellular dynamics of inoculated PrP-res and newly generated PrPSc during early stage prion infection in Neuro2a cells. Virology.

[295] Telling G.C., Scott M., Mastrianni J., Gabizon R., Torchia M., Cohen F.E., DeArmond S.J., Prusiner S.B. (1995). Prion propagation in mice expressing human and chimeric PrP transgenes implicates the interaction of cellular PrP with another protein. Cell.

[296] Basler K., Oesch B., Scott M., Westaway D., Walchli M., Groth D.F., McKinley M.P., Prusiner S.B., Weissmann C. (1986). Scrapie and cellular PrP isoforms are encoded by the same chromosomal gene. Cell.

[297] Riek R., Hornemann S., Wider G., Billeter M., Glockshuber R., Wuthrich K. (1996). NMR structure of the mouse prion protein domain PrP (121–231). Nature.

[298] Taraboulos A., Scott M., Semenov A., Avrahami D., Laszlo L., Prusiner S.B. (1995). Cholesterol depletion and modification of COOH-terminal targeting sequence of the prion protein inhibit formation of the scrapie isoform. J. Cell Biol..

[299] Lopes M.H., Hajj G.N., Muras A.G., Mancini G.L., Castro R.M., Ribeiro K.C., Brentani R.R., Linden R., Martins V.R. (2005). Interaction of cellular prion and stress-inducible protein 1 promotes neuritogenesis and neuroprotection by distinctsignaling pathways. J. Neurosci..

[300] Zanata S.M., Lopes M.H., Mercadante A.F., Hajj G.N., Chiarini L.B., Nomizo R., Freitas A.R., Cabral A.L., Lee K.S., Juliano M.A. (2002). Stress-inducible protein 1 is a cell surface ligand for cellular prion that triggersneuroprotection. EMBO J..

[301] Santuccione A., Sytnyk V., Leshchyns’ka I., Schachner M. (2005). Prion proteinrecruits its neuronal receptor NCAM to lipid rafts to activate p59fyn and toenhance neurite outgrowth. J. Cell Biol..

[302] Schmitt-Ulms G., Legname G., Baldwin M.A., Ball H.L., Bradon N., Bosque P.J., Crossin K.L., Edelman G.M., DeArmond S.J., Cohen F.E. (2001). Binding of neural cell adhesion molecules (*N*-CAMs) to the cellular prion protein. J. Mol. Biol..

[303] Brown D.R., Qin K., Herms J.W., Madlung A., Manson J., Strome R., Fraser P.E., Kruck T., von Bohlen A., Schulz-Schaeffer W. (1997). The cellular prion protein binds copper *in vivo*. Nature.

[304] Jones C.E., Abdelraheim S.R., Brown D.R., Viles J.H. (2004). Preferential Cu^2+^ coor-dination by His96 and His111 induces beta-sheet formation in the unstructuredamyloidogenic region of the prion protein. J. Biol. Chem..

[305] Zeng L., Zou W., Wang G. (2015). Cellular prion protein (PrP(C)) and its role in stress responses. Int. J. Clin. Exp. Med..

[306] Yang D.S., Stavrides P., Mohan P.S., Kaushik S., Kumar A., Ohno M., Schmidt S.D., Wesson D., Bandyopadhyay U., Jiang Y. (2011). Reversal of autophagy dysfunction in the TgCRND8 mouse model of Alzheimer’s disease ameliorates amyloid pathologies and memory deficits. Brain.

[307] Thackray A.M., Knight R., Haswell S.J., Bujdoso R., Brown D.R. (2002). Metal imbalance and compromised antioxidant function are early changes in prion disease. Biochem. J..

[308] Didonna A. (2013). Prion protein and its role in signal transduction. Cell. Mol. Biol. Lett..

[309] Martins V.R., Beraldo F.H., Hajj G.N., Lopes M.H., Lee K.S., Prado M.A., Linden R. (2010). Prion protein: Orchestrating neurotrophic activities. Curr. Issues Mol. Biol..

[310] Sunyach C., Jen A., Deng J., Fitzgerald K.T., Frobert Y., Grassi J., McCaffrey M.W., Morris R. (2003). The mechanism of internalization of glycosylphosphatidylinositol-anchored prion protein. EMBO J..

[311] Campana V., Sarnataro D., Zurzolo C. (2005). The highways and byways of prionprotein trafficking. Trends Cell Biol..

[312] Ashok A., Hegde R.S. (2008). Retrotranslocation of prion proteins from the endo-plasmic reticulum by preventing GPI signal transamidation. Mol. Biol. Cell.

[313] Yedidia Y., Horonchik L., Tzaban S., Yanai A., Taraboulos A. (2001). Proteasomesand ubiquitin are involved in the turnover of the wild-type prion protein. EMBOJ..

[314] Drisaldi B., Stewart R.S., Adles C., Stewart L.R., Quaglio E., Biasini E., Fior-iti L., Chiesa R., Harris D.A. (2003). Mutant PrP is delayed in its exit fromthe endoplasmic reticulum, but neither wild-type nor mutant PrP under-goes retrotranslocation prior to proteasomal degradation. J. Biol. Chem..

[315] Orsi A., Fioriti L., Chiesa R., Sitia R. (2006). Conditions of endoplasmic reticulumstress favor the accumulation of cytosolic prion protein. J. Biol. Chem..

[316] Levine C.G., Mitra D., Sharma A., Smith C.L., Hegde R.S. (2005). The efficiency ofprotein compartmentalization into the secretory pathway. Mol. Biol. Cell.

[317] Rane N.S., Kang S.W., Chakrabarti O., Feigenbaum L., Hegde R.S. (2008). Reduced translocation of nascent prion protein during ER stress contributes to neurodegeneration. Dev. Cell.

[318] Roucou X., Guo Q., Zhang Y., Goodyer C.G., Le Blanc A.C. (2003). Cytosolic prion pro-tein is not toxic and protects against Bax-mediated cell death in human primaryneurons. J. Biol. Chem..

[319] Roucou X., Gains M., LeBlanc A.C. (2004). Neuroprotective functions of prion protein. J. Neurosci. Res..

[320] Roucou X., Giannopoulos P.N., Zhang Y., Jodoin J., Goodyer C.G., LeBlanc A. (2005). Cellular prion protein inhibits proapoptotic Bax conformational changein human neurons and in breast carcinoma MCF-7 cells. Cell Death Differ..

[321] Vanderperre B., Staskevicius A.B., Tremblay G., McCoy M., O’Neill M.A., Cashman N.R., Roucou X. (2011). An overlapping reading frame in the PRNP gene encodesa novel polypeptide distinct from the prion protein. FASEB J..

[322] Milhavet O., Lehmann S. (2002). Oxidative stress and the prion protein in transmissible spongiform encephalopathies. Brain Res. Brain Res. Rev..

[323] Brown D.R. (2005). Neurodegeneration and oxidative stress: Prion disease results from loss of antioxidant defence. Folia Neuropathol..

[324] Nadal R.C., Abdelraheim S.R., Brazier M.W., Rigby S.E., Brown D.R., Viles J.H. (2007). Prion protein does not redox-silence Cu^2+^, but is a sacrificial quencher of hydroxyl radicals. Free Radic. Biol. Med..

[325] Stadtman E.R. (2001). Protein oxidation in aging and age-related diseases. Ann. N. Y. Acad. Sci..

[326] Cui Z.J., Han Z.Q., Li Z.Y. (2012). Modulating protein activity and cellular function by methionine residue oxidation. Amino Acids.

[327] Moskovitz J., Bar-Noy S., Williams W.M., Requena J., Berlett B.S., Stadtman E.R. (2001). Methionine sulfoxide reductase (MsrA) is a regulator of antioxidant defense and lifespan in mammals. Proc. Natl. Acad. Sci. USA.

[328] Elmallah M.I., Borgmeyer U., Betzel C., Redecke L. (2013). Impact of methionine oxidation as an initial event on the pathway of human prion protein conversion. Prion.

[329] Younan N.D., Nadal R.C., Davies P., Brown D.R., Viles J.H. (2012). Methionine oxidation perturbs the structural core of the prion protein and suggests a generic misfolding pathway. J. Biol. Chem..

[330] Caughey B.W., Dong A., Bhat K.S., Ernst D., Hayes S.F., Caughey W.S. (1991). Secondary structure analysis of the scrapie-associated protein PrP 27–30 in water by infrared spectroscopy. Biochemistry.

[331] Abid K., Soto C. (2006). The intriguing prion disorders. Cell Mol. Life Sci..

[332] Thellung S., Corsaro A., Villa V., Simi A., Vella S., Pagano A., Florio T. (2011). Human PrP90–231-induced cell death is associated with intracellular accumulation of insoluble and protease-resistant macroaggregates and lysosomal dysfunction. Cell Death Dis..

[333] Chiovitti K., Corsaro A., Thellung S., Villa V., Paludi D., D’Arrigo C., Russo C., Perico A., Ianieri A., di Cola D. (2007). Intracellular accumulation of a mild-denatured monomer of the human PrP fragment 90–231, as possible mechanism of its neurotoxic effects. J. Neurochem..

[334] Borger E., Aitken L., Muirhead K.E., Allen Z.E., Ainge J.A., Conway S.J., Gunn-Moore F.J. (2011). Mitochondrial beta-amyloid in Alzheimer’s disease. Biochem. Soc. Trans..

[335] Coskun P., Wyrembak J., Schriner S.E., Chen H.W., Marciniack C., LaFerla F., Wallace D.C. (2012). A mitochondrial etiology of Alzheimer and Parkinson disease. Biochim. Biophys. Acta.

[336] Siskova Z., Mahad D.J., Pudney C., Campbell G., Cadogan M., Asuni A., O’Connor V., Perry V.H. (2010). Morphological and functional abnormalities in mitochondria associated with synaptic degeneration in prion disease. Am. J. Pathol..

[337] Corsaro A., Thellung S., Villa V., Nizzari M., Florio T. (2012). Role of prion protein aggregation in neurotoxicity. Int. J. Mol. Sci..

[338] Belay E.D. (1999). Transmissible spongiform encephalopathies in humans. Annu. Rev. Microbiol..

[339] Hetz C., Russelakis-Carneiro M., Wälchli S., Carboni S., Vial-Knecht E., Maundrell K., Castilla J., Soto C. (2005). The disulfide isomerase Grp58 is a protective factor against prion neurotoxicity. J. Neurosci..

[340] Hetz C., Russelakis-Carneiro M., Maundrell K., Castilla J., Soto C. (2003). Caspase-12 and endoplasmic reticulum stress mediate neurotoxicity of pathological prion protein. EMBO J..

[341] Hetz C., Lee A.H., Gonzalez-Romero D., Thielen P., Castilla J., Soto C., Glimcher L.H. (2008). Unfolded protein response transcription factor XBP-1 does not influence prion replication or pathogenesis. Proc. Natl. Acad. Sci. USA.

[342] Steele A.D., Hetz C., Yi C.H., Jackson W.S., Borkowski A.W., Yuan J., Wollmann R.H., Lindquist S. (2007). Prion pathogenesis is independent of caspase-12. Prion.

[343] Saleh M., Mathison J.C., Wolinski M.K., Bensinger S.J., Fitzgerald P., Droin N., Ulevitch R.J., Green D.R., Nicholson D.W. (2006). Enhanced bacterial clearance and sepsis resistance in caspase-12-deficient mice. Nature.

[344] Saleh M., Vaillancourt J.P., Graham R.K., Huyck M., Srinivasula S.M., Alnemri E.S., Steinberg M.H., Nolan V., Baldwin C.T., Hotchkiss R.S. (2004). Differential modulation of endotoxin responsiveness by human caspase-12 polymorphisms. Nature.

[345] Moreno J.A., Halliday M., Molloy C., Radford H., Verity N., Axten J.M., Ortori C.A., Willis A.E., Fischer P.M., Barrett D.A., Mallucci G.R. (2013). Oral treatment targeting the unfolded protein response prevents neurodegeneration and clinical disease in prion-infected mice. Sci. Transl. Med..

[346] Nunziante M., Ackermann K., Dietrich K., Wolf H., Gädtke L., Gilch S., Vorberg I., Groschup M., Schätzl H.M. (2011). Proteasomal dysfunction and endoplasmic reticulum stress enhance traffi cking of prion protein aggregates through the secretory pathway and increase accumulation of pathologic prion protein. J. Biol. Chem..

[347] Boellaard J.W., Kao M., Schlote W., Diringer H. (1991). Neuronal autophagy in experimental scrapie. Acta Neuropathol..

[348] Boellaard J.W., Schlote W., Tateishi J. (1989). Neuronal autophagy in experimental Creutzfeldt-Jakob’s disease. Acta Neuropathol..

[349] Schatzl H.M., Laszlo L., Holtzman D.M., Tatzelt J., DeArmond S.J., Weiner R.I., Mobley W.C., Prusiner S.B. (1997). A hypothalamic neuronal cell line persistently infected with scrapie prions exhibits apoptosis. J. Virol..

[350] Liberski P.P., Sikorska B., Bratosiewicz-Wasik J., Gajdusek D.C., Brown P. (2004). Neuronal cell death in transmissible spongiform encephalopathies (prion diseases) revisited: From apoptosis to autophagy. Int. J. Biochem. Cell Biol..

[351] Sikorska B., Liberski P.P., Giraud P., Kopp N., Brown P. (2004). Autophagy is a part of ultrastructural synaptic pathology in Creutzfeldt-Jakob disease: A brain biopsy study. Int. J. Biochem. Cell Biol..

[352] Dandoy-Dron F., Benboudjema L., Guillo F., Jaegly A., Jasmin C., Dormont D., Tovey M.G., Dron M. (2000). Enhanced levels of scrapie responsive gene mRNA in BSE-infected mouse brain. Brain Res..

[353] Dandoy-Dron F., Guillo F., Benboudjema L., Deslys J.P., Lasmezas C., Dormont D., Tovey M.G., Dron M. (1998). Gene expression in scrapie. Cloning of a new scrapie-responsive gene and the identification of increased levels of seven other mRNA transcripts. J. Biol. Chem..

[354] Dron M., Dandoy-Dron F., Guillo F., Benboudjema L., Hauw J.J., Lebon P., Dormont D., Tovey M.G. (1998). Characterization of the human analogue of a Scrapie-responsive gene. J. Biol. Chem..

[355] Dron M., Bailly Y., Beringue V., Haeberle A.M., Griffond B., Risold P.Y., Tovey M.G., Laude H., Dandoy-Dron F. (2005). Scrg1 is induced in TSE and brain injuries, and associated with autophagy. Eur. J. Neurosci..

[356] Dron M., Bailly Y., Beringue V., Haeberle A.M., Griffond B., Risold P.Y., Tovey M.G., Laude H., Dandoy-Dron F. (2006). SCRG1, a potential marker of autophagy in transmissible spongiform encephalopathies. Autophagy.

[357] Oh J.M., Shin H.Y., Park S.J., Kim B.H., Choi J.K., Choi E.K., Carp R.I., Kim Y.S. (2008). The involvement of cellular prion protein in the autophagy pathway in neuronal cells. Mol. Cell Neurosci..

[358] Liberski P.P., Brown D.R., Sikorska B., Caughey B., Brown P. (2008). Cell death and autophagy in prion diseases (transmissible spongiform encephalopathies). Folia Neuropathol.

[359] Liberski P.P., Gajdusek D.C., Brown P. (2002). How do neurons degenerate in prion diseases or transmissible spongiform encephalopathies (TSEs): Neuronal autophagy revisited. Acta Neurobiol. Exp..

[360] Mok S.W., Riemer C., Madela K., Hsu D.K., Liu F.T., Gultner S., Heise I., Baier M. (2007). Role of galectin-3 in prion infections of the CNS. Biochem. Biophys. Res. Commun..

[361] Ertmer A., Gilch S., Yun S.W., Flechsig E., Klebl B., Stein-Gerlach M., Klein M.A., Schatzl H.M. (2004). The tyrosine kinase inhibitor STI571 induces cellular clearance of PrPSc in prion-infected cells. J. Biol. Chem..

[362] Ertmer A., Huber V., Gilch S., Yoshimori T., Erfle V., Duyster J., Elsasser H.P., Schatzl H.M. (2007). The anticancer drug imatinib induces cellular autophagy. Leukemia.

[363] Yun S.W., Ertmer A., Flechsig E., Gilch S., Riederer P., Gerlach M., Schatzl H.M., Klein M.A. (2007). The tyrosine kinase inhibitor imatinib mesylate delays prion neuroinvasion by inhibiting prion propagation in the periphery. J. Neurovirol..

[364] Aguib Y., Gilch S., Krammer C., Ertmer A., Groschup M.H., Schatzl H.M. (2008). Neuroendocrine cultured cells counteract persistent prion infection by downregulation of PrPc. Mol. Cell Neurosci..

[365] Heiseke A., Schobel S., Lichtenthaler S.F., Vorberg I., Groschup M.H., Kretzschmar H., Schatzl H.M., Nunziante M. (2008). The novel sorting nexin SNX33 interferes with cellular PrP formation by modulation of PrP shedding. Traffic.

[366] Marella M., Lehmann S., Grassi J., Chabry J. (2002). Filipin prevents pathological prion protein accumulation by reducing endocytosis and inducing cellular PrP release. J. Biol. Chem..

[367] Parkin E.T., Watt N.T., Turner A.J., Hooper N.M. (2004). Dual mechanisms for shedding of the cellular prion protein. J. Biol. Chem..

[368] Magalhaes A.C., Baron G.S., Lee K.S., Steele-Mortimer O., Dorward D., Prado M.A.M., Caughey B. (2005). Uptake and neuritic transport of scrapie prion protein coincident with infection of neuronal cells. J. Neurosci..

[369] Rouvinski A., Karniely S., Kounin M., Moussa S., Goldberg M.D., Warburg G., Lyakhovetsky R., Papy-Garcia D., Kutzsche J., Korth C. (2014). Live imaging of prions reveals nascent PrPSc in cell-surface, raft-associated amyloid strings and webs. J. Cell Biol..

[370] Veith N.M., Plattner H., Stuermer C.A.O., Schulz-Schaeffer W.J., Burkle A. (2009). Immunolocalisation of PrPSc in scrapie-infected N2a mouse neuroblastoma cells by light and electron microscopy. Eur. J. Cell Biol..

[371] Yao H., Zhao D.M., Khan S.H., Yang L.F. (2013). Role of autophagy in prion proteininduced neurodegenerative diseases. Acta Biochim. Biophys. Sin..

[372] Ashok A., Hegde R.S. (2009). Selective processing and metabolism of disease-causing mutant prion proteins. PLoS Pathog..

[373] Goold R., McKinnon C., Rabbanian S., Collinge J., Schiavo G., Tabrizi S.J. (2013). Alternative fates of newly formed PrPSc upon prion conversion on the plasma membrane. J. Cell Sci..

[374] Goold R., McKinnon C., Tabrizi S.J. (2015). Prion degradation pathways: Potential for therapeutic intervention. Mol. Cell Neurosci..

[375] Grenier C., Bissonnette C., Volkov L., Roucou X. (2006). Molecular morphology and toxicity of cytoplasmic prion protein aggregates in neuronal and non-neuronal cells. J. Neurochem..

[376] Kristiansen M., Messenger M.J., Klohn P.C., Brandner S., Wadsworth J.D.F., Collinge J., Tabrizi S.J. (2005). Disease-related prion protein forms aggresomes in neuronal cells leading to caspase activation and apoptosis. J. Biol. Chem..

[377] Micsenyi M.C., Sikora J., Stephney G., Dobrenis K., Walkley S.U. (2013). Lysosomal membrane permeability stimulates protein aggregate formation in neurons of a lysosomal disease. J. Neurosci..

[378] Jin T.C., Gu Y.P., Zanusso G., Sy M.S., Kumar A., Cohen M., Gambetti P., Singh N. (2000). The chaperone protein BiP binds to amutant prion protein and mediates its degradation by the proteasome. J. Biol. Chem..

[379] Zanusso G., Petersen R.B., Jin T.C., Jing Y., Kanoush R., Ferrari S., Gambetti P., Singh N. (1999). Proteasomal degradation and *N*-terminal protease resistance of the codon 145 mutant prion protein. J. Biol. Chem..

